# Core–sheath coupling controls flagellar curvature and motility in *Leptospira*

**DOI:** 10.1038/s41467-026-75995-6

**Published:** 2026-07-25

**Authors:** Fabiana San Martin, Megan R. Brady, Lenka Fule, Lucienne Nouchikian, Azalia Rodriguez, Magalie Duchateau, Sonia Mondino, Nicole Larrieux, Elsio A. Wunder, Albert I. Ko, Martial Rey, Julia Chamot-Rooke, Rosario Duran, Felipe Trajtenberg, Mathieu Picardeau, Charles V. Sindelar, Alejandro Buschiazzo

**Affiliations:** 1 Institut Pasteur de Montevideo, Laboratory of Molecular & Structural Microbiology, Montevideo, Uruguay; 2 Department of Molecular Biophysics and Biochemistry, Yale University, New Haven, CT USA; 3 Institut Pasteur, Université Paris Cité, Biology of Spirochetes Unit, Paris, France; 4 Institut Pasteur, Université Paris Cité, CNRS UAR 2024, Mass Spectrometry for Biology Unit, Paris, France; 5 Institut Pasteur de Montevideo & Instituto de Investigaciones Biologicas Clemente Estable, Analytical Biochemistry and Proteomics Unit, Montevideo, Uruguay; 6 Department of Epidemiology of Microbial Diseases, Yale School of Public Health, New Haven, CT USA; 7 Gonçalo Moniz Institute, Oswaldo Cruz Foundation, Brazilian Ministry of Health, Salvador, Brazil; 8 Locbio, Montevideo, Uruguay; 9 Institut Pasteur, Department of Microbiology, Paris, France; 10 Present Address: Exton, PA USA; 11 Present Address: Institute of Biology, Humboldt-Universität zu Berlin, Berlin, Germany; 12 Present Address: AbbVie Deutschland GmbH & Co. KG, Analytical Research & Development, Knollstraße, Ludwigshafen am Rhein, Germany; 13 Present Address: Department of Pathobiology and Veterinary Science, University of Connecticut, Storrs, CT USA; 14 Present Address: Physiology and Cancer Department, Institut de Génomique Fonctionnelle, Université de Montpellier, Montpellier, France; 15 Present Address: Yale Center for Research Computing, Yale University, New Haven, CT USA

**Keywords:** Bacterial structural biology, Pathogens, Cryoelectron microscopy, Bacterial genetics

## Abstract

Spirochaete pathogens are among the most invasive bacteria known, causing syphilis, Lyme disease, and leptospirosis. Their tissue penetration depends on periplasmic flagellar filaments that, unlike other bacterial flagella, are encased in a spirochaete-specific multi-protein sheath and deform the cell body into motile waves. How these filaments achieve the mechanical properties needed for invasive motility has remained unclear. Here we determine complete atomic structures of the *Leptospira* endoflagellar filament, revealing an elaborate sheath of 9 to 12 distinct asymmetrically arranged proteins. We show that the flagellin variant forming the filament core determines sheath composition, producing curvatures ranging from ~3.5 µm^−1^ to ~5.6 µm^−1^. The lower-curvature architecture, employed by pathogenic *Leptospira interrogans*, proves essential for motility in viscous environments and during infection. Thus, *Leptospira* achieves environment-specific motility through modular core–sheath coupling, linking atomic-scale structural plasticity to large-scale changes in swimming behaviour. Conservation of key sheath components suggests this mechanism may extend across spirochaetes.

## Abstract (web summary)

Pathogenic spirochaetes have periplasmic flagella that give these bacteria their spiral shape and enable penetration into host tissues. Here, San Martin et al. present detailed structures of the flagellar filaments and their multi-protein sheath, linking structural plasticity to motility changes.

## Introduction

Spirochaete bacteria encompass some of the most invasive bacterial pathogens known^1^, including the causative agents of leptospirosis (*Leptospira* spp.), syphilis (*Treponema pallidum*), and Lyme disease (*Borrelia burgdorferi*). Their ability to rapidly disseminate through connective tissue, blood, and organs, including crossing biological barriers like the blood-brain barrier, relies on a distinctive swimming mechanism powered by periplasmic flagella (endoflagella). Unlike conventional bacterial flagella that extend freely into the aqueous medium^2^, spirochaete endoflagellar filaments are confined within the periplasm, where they transmit motor-generated torque along micron-scale lengths to deform the spiral-shaped cell body^3,4^. Spirochaete flagellar filaments precisely tune curvature and stiffness to enable motility across environments spanning orders of magnitude in viscosity, from fluids to host tissues^3,5^.

Spirochaete pathogens cause serious zoonoses, vector-borne diseases, and sexually transmitted infections that affect millions worldwide each year. Among spirochaetes, pathogenic *Leptospira* species are estimated to cause more than 1 million cases of severe leptospirosis and nearly 60,000 deaths annually^6^. This globally important zoonosis is usually transmitted through contact with water contaminated with urine from animal reservoirs^7^. The disease ranges from mild self-limiting illness to severe manifestations including haemorrhage, jaundice, and renal failure (Weil’s disease). Beyond its medical relevance, *Leptospira* offers a powerful system for dissecting structure-function relationships in spirochaete motility^8^: it possesses a single flagellum at each cell pole, providing structural simplicity compared to other spirochaetes. The saprophytic model *L. biflexa* enables rapid culture, high biomass yields, and tractable genetic manipulation. This combination enables detailed structural studies and direct comparison between pathogenic and non-pathogenic species.

Understanding flagellar structure is critical because spirochaete motility is a key determinant of virulence. *Leptospira* mutants lacking functional flagella show severely attenuated infection in animal models^9,10^, and spirochetal motility is linked to distinctive features of their flagellar motor (including a periplasmic collar and additional stators), hook (with extensive inter-subunit cross-linking of FlgE), and filament (the proteinaceous sheath of additional proteins producing curved, stiff assemblies), collectively thought to support the higher torque loads typical of spirochaete swimming^11,12^. Of these features, the filament sheath has no counterpart in exoflagellated bacteria, whose filaments consist of flagellin alone^3^. Until recently, the only known sheath component was FlaA, conserved across the phylum^3^; however, the discovery of two additional, *Leptospira*-specific sheath components (FcpA and FcpB) revealed that sheath composition was more complex^10,13^. Structural studies of *Leptospira* filaments by cryo-electron tomography identified an asymmetric, uneven distribution of sheath proteins covering the flagellin core in native supercoiled configurations^14^. Subsequently, high-resolution structures of mutant *Leptospira* filaments uncovered the role that individual sheath components (FlaA2, FcpA and FcpB) play to induce filament supercoiling, demonstrating that an asymmetric protein arrangement is essential for flagellar function^15^. However, how the complete sheath is organized in wild-type filaments, and how it cooperates with the flagellin core to control filament curvature and motility, have remained unknown.

Here we present complete atomic structures of endoflagellar filaments from *L. biflexa* and from the pathogen *L. interrogans*, integrating cryo-electron microscopy (cryo-EM), quantitative and crosslinking mass spectrometry (MS), and X-ray crystallography. We show that the sheath architecture is more elaborate than previously anticipated, with 9 to 12 distinct proteins asymmetrically enveloping the flagellin core. Furthermore, comparison of the wild-type pathogen cryo-EM structure with that of a *flaB1*^*-*^ knockout mutant (deleting the main core flagellin) reveals that swapping core isoforms (from FlaB1 to FlaB4) reorganises the sheath and substantially increases the filament curvature, abrogating virulence and tissue penetration. These findings reveal how modular core-sheath coupling links atomic-scale structural plasticity to large-scale flagellar mechanics and invasive motility.

## Results

### *Leptospira biflexa* filaments display a highly elaborate sheath organization

**Fig. 1:**
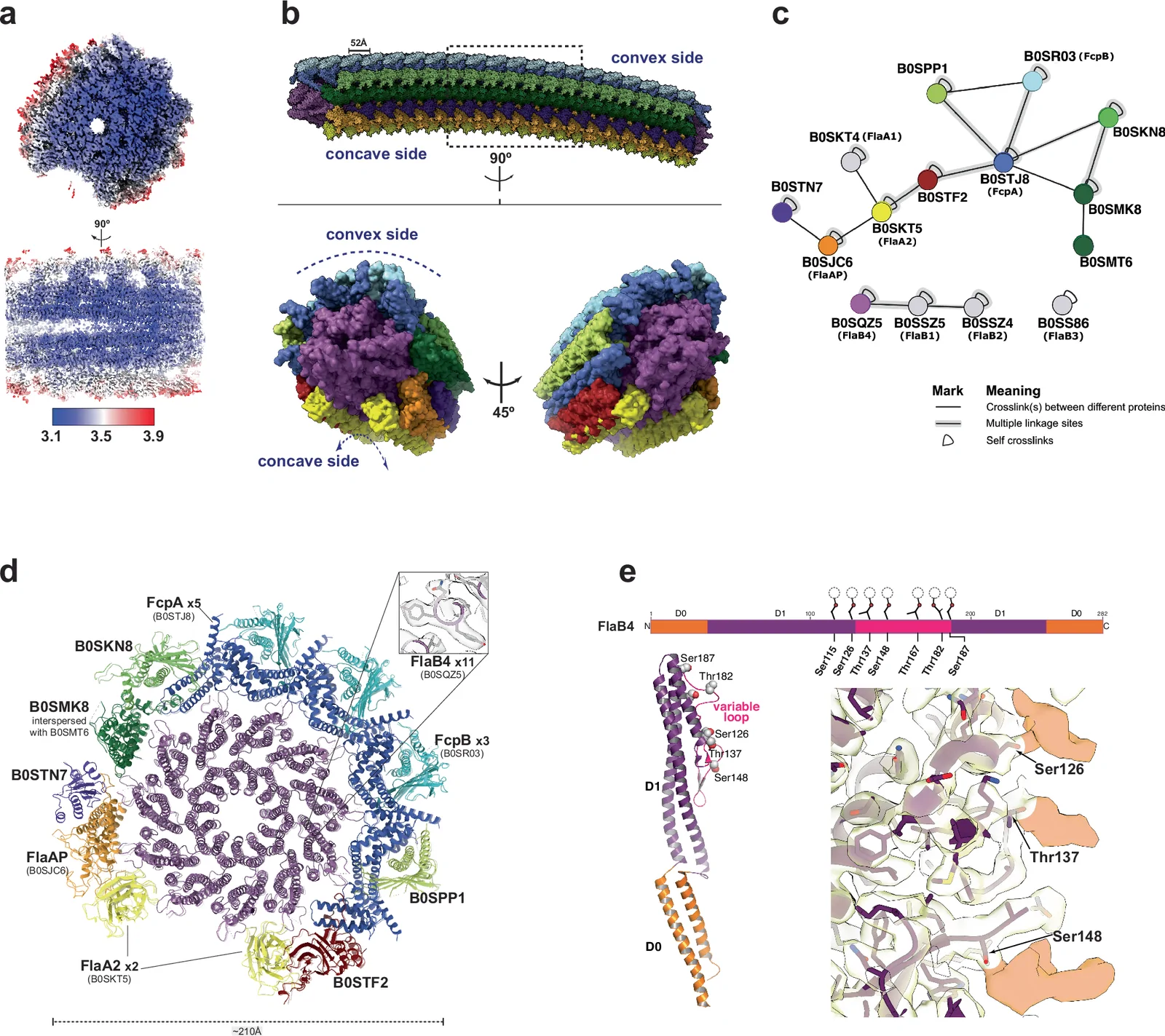
Integrative 3D structure determination of *Leptospira biflexa* flagellar filaments. **a** Cryo-EM map of a native *L. biflexa* filament coloured by local resolution (scale bar: 3.1-3.9 Å), viewed from one end (top) and from the side (bottom), contoured at 0.05 threshold. **b** Cryo-EM reconstruction shown as a molecular surface, coloured by protein identity. Top: side view with convex and concave sides indicated; dashed box marks the size of the reconstructed volume (~7 repeat layers), extended at both ends by superposition of terminal layers to visualize overall curvature more clearly. Bottom: end-on views of the refined volume, rotated 45° relative to each other to reveal lateral sheath organisation surrounding the FlaB4 core. **c** Crosslinking mass spectrometry interaction network, created with xiView^61^ (https://www.xiview.org). Nodes represent identified filament proteins, coloured as in panels (b,d) and labelled with UniProt IDs (gene names in parentheses for annotated proteins). Edges indicate inter-protein crosslinks; thick edges denote multiple linkage sites; self-crosslinks are shown as loops. Core flagellins (bottom) lack inter-protein crosslinks with sheath components, consistent with glycan-mediated core-sheath interactions. An interactive version of this network is publicly accessible at https://www.xiview.org/network.php?upload=42923-71803-52415-45391-89719, and allows users to explore individual crosslinks and protein properties. **d** Refined atomic model shown as cartoon, viewed as a transverse cross-section representing one helical axial repeat (~52 Å). Proteins labelled with UniProt IDs and copy numbers per axial repeat. Inset: FlaB4 core cryo-EM density (semi-transparent surface) with fitted atomic model (sticks). **e** Schematic of FlaB4 sequence (282 residues, drawn to scale), shaded by domain (D0, D1, central variable loop), showing the position of Ser/Thr residues bearing visible *O*-glycosylation density in the cryo-EM map. Left: ribbon model of one FlaB4 protomer, with the seven modified Ser/Thr sites shown as Cα spheres (only five labelled for clarity). Right: representative cryo-EM map (yellow mesh) around three of the modified residues on one FlaB4 protomer (purple cartoon), with the corresponding *O*-linked carbohydrate moieties’ densities highlighted in orange.

Cryo-EM structures of wild-type (*wt*) *Leptospira biflexa* were determined at 3.5 Å resolution^16^. Density modification^17^ improved map details, achieving a final resolution of 3.2 Å. (Fig. 1a). The atomic structure (Fig. 1b) was solved using an integrative strategy (Supplementary Fig. 1) that combined cryo-EM single-particle analysis (Supplementary Table 1; Supplementary Fig. 2), crosslinking and quantitative MS (Table 1; Fig. 1c; Supplementary Fig. 3; Supplementary Data 1, 2), X-ray crystallographic structures of FcpA and FcpB^14^, and AlphaFold3 predictions. This process was iterated to obtain the final structure: comparative proteomics identified candidate proteins including unannotated gene products, crosslinking data prioritized these by proximity^18^, and cryo-EM density fitting provided validation. Each confirmed component enabled re-interrogation of crosslinking interaction networks, progressively expanding the catalogue of filament constituents.

**Table 1:**
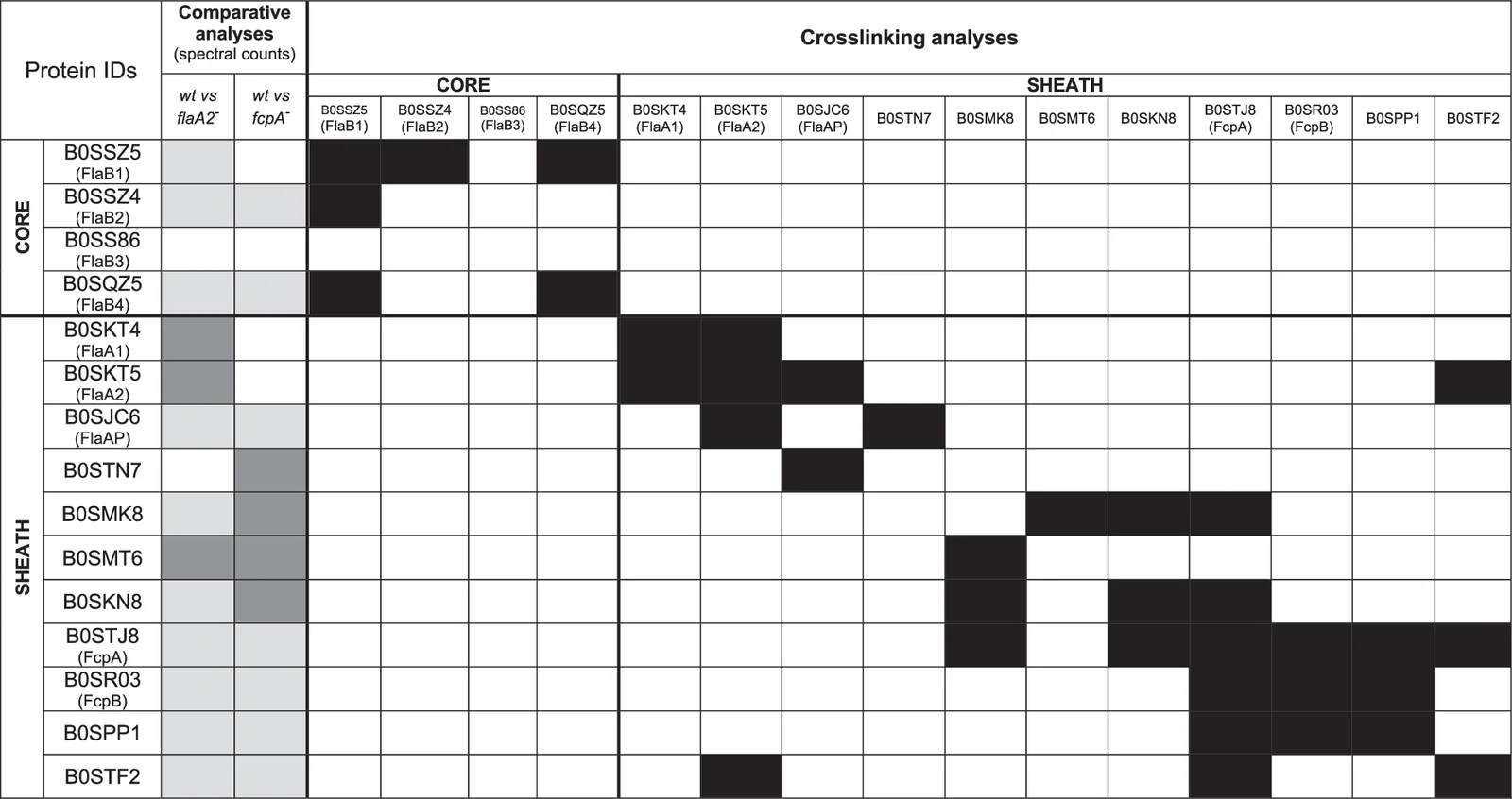
Proteins identified by comparative proteomics and crosslinking mass spectrometry analyses of *Leptospira biflexa* flagellar filaments

When within the same protein species, crosslinks between different subunits in the filament are shown (inter-crosslinks)

See Supplementary Data 1 & 2 for full details; and https://www.xiview.org/network.php?upload=42923-71803-52415-45391-89719 for interactive analysis.

Shading colour scheme:

Exclusive: proteins detected in one condition but below the detection threshold in the other

Fold Change >1.5 (*wt* over mutant)

Crosslinked

The refined *L. biflexa* filament model comprises a core of flagellin (FlaB) protomers, polymerized helically (twist 65.4°; rise 4.73 Å) into 11 protofilaments enclosing a ~20 Å central channel (Fig. 1b,d). This regular helical packing imprints the core with an axial repeat of ~52 Å (11 × 4.73 Å). Inter-protomer contacts are established along three principal subsidiary helices: the 11-start (corresponding to the longitudinal protofilaments), the right-handed 6-start, and the left-handed −5-start^19^. Although *Leptospira* expresses four FlaB isoforms^20^, *L. biflexa wt* filaments display FlaB4 as the most abundant one (Supplementary Data 3), with distinctive side chains and *O*-glycosylation sites visible in the cryo-EM density (Fig. 1e).

The flagellin core is coated with 12 distinct sheath proteins (all but one assigned to specific identities by our integrative analysis). The sheath components are arranged around the filament in a radially asymmetric organisation, and overall tracking the ~52 Å helical repeat of the core (Fig. 1b,d). This architecture starkly contrasts with previously reported, core-only bacterial flagellar filament structures, and more than doubles the known number of sheath components in spirochaetes, including *Leptospira*.

The sheath proteins bind to specific core protofilaments around the filament circumference, giving each side of the assembly distinct features (Fig. 1d; Supplementary Fig. 4; Supplementary Table 2). The sheath is overall organized in two concentric tiers around the core. The inner tier establishes direct contacts with FlaB, primarily through glycans^15^: FlaA2 moieties coat the concave surface, each associated with distinct partner proteins, FlaAP on one side and B0STF2 on the other; FcpA protomers line the convex surface; and a mixed protofilament of alternating B0SMK8 and B0SMT6 (structurally similar to FlaAP and showing inter-crosslinks between them by MS, see Fig. 1c) completes the core-contacting shell. An outer tier caps this first layer without direct core contact: FcpB moieties sit atop FcpA on the convex side, as do B0SPP1 and B0SKN8 (which share an FcpB-like fold); B0STN7 caps the junction between FlaAP and B0SMK8; one additional outer-tier component, capping FlaA2 and FlaAP, is visible in the cryo-EM map but could not be assigned to a specific protein (Supplementary Fig. 4d). Complementary ridges and troughs at the core-sheath interface, with helical pseudosymmetry matching the core’s, mediate these contacts throughout. Notably, a groove remains at the concave surface between the two FlaA2 protofilaments, as reported in earlier tomography studies^14^. Although MS data unambiguously detected FlaA1 in purified samples, including crosslinks to FlaA2 (Fig. 1c; Table 1), and the groove could accommodate two FlaA1 units per sheath layer, no cryo-EM density could be attributed to this protein, suggesting it progressively dissociates during sample handling, and prompting further investigation of this poorly characterized sheath component.

### FlaA1 structure reveals a new Carbohydrate Binding Module family and calcium-dependent stability

**Fig. 2:**
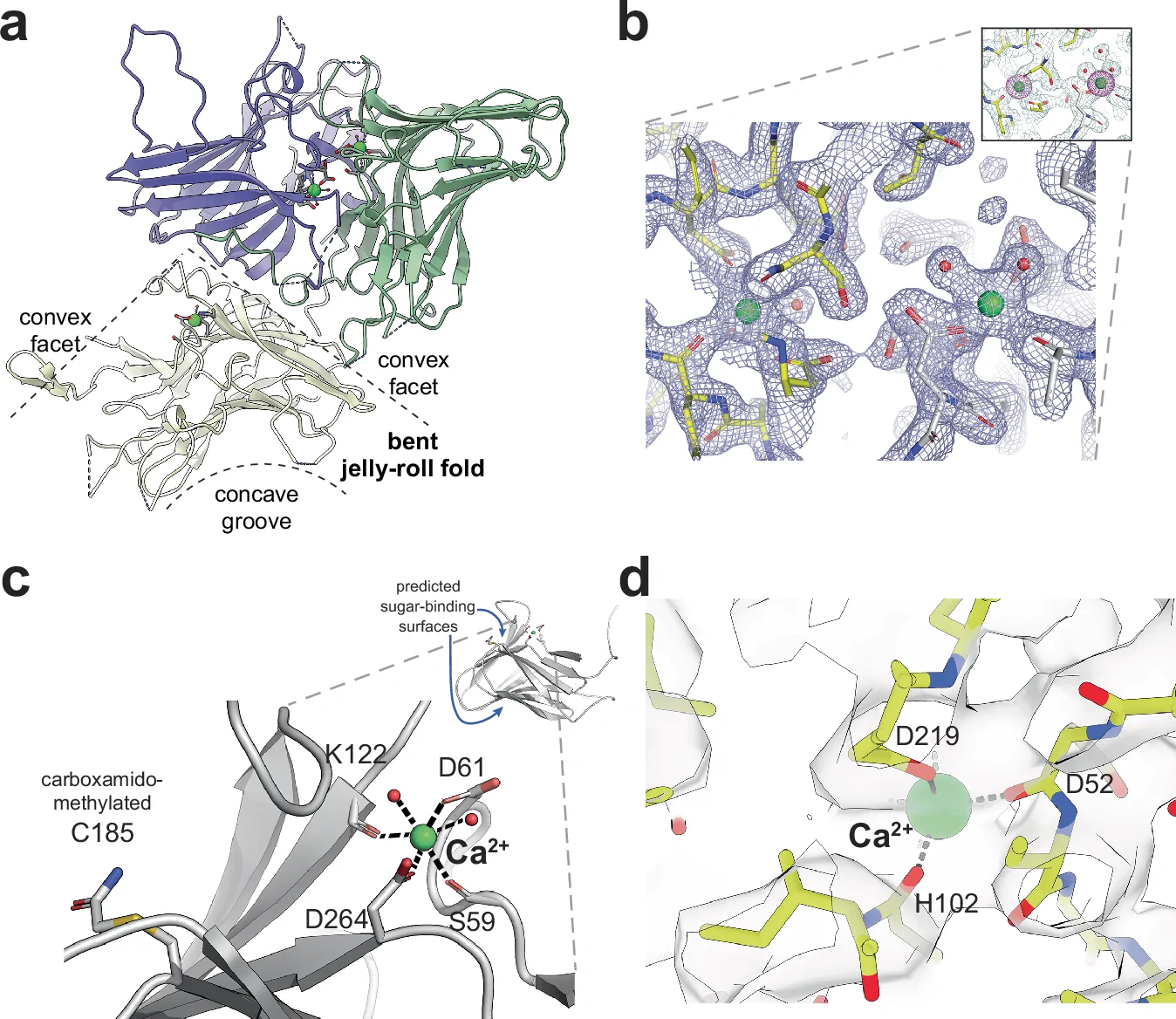
FlaA1 structure reveals a calcium-dependent carbohydrate-binding module. **a** Crystal structure of *L. borgpetersenii* FlaA1 at 1.82 Å resolution. The asymmetric unit contains three monomers shown as cartoons in different colours. Ca^2+^ ions are shown as green spheres. The jelly-roll-like β-sandwich fold is highlighted, with a concave groove and opposite convex facets. **b** Representative σA-weighted 2mFo-DFc electron density map contoured at 1.2σ, showing well-resolved Ca^2+^ coordination and surrounding residues. Inset: mFo−DFc omit map (magenta mesh, contoured at +5σ) calculated with the calcium ions removed from the model, confirming the assignment without model bias; the 2mFo−DFc map is overlaid for reference (cyan mesh). **c** Close-up of the Ca^2+^-binding site showing octahedral coordination by Asp61 and Asp264 (side chain oxygens), Ser59 and Lys122 (backbone carbonyls), and two water molecules. The carboxamidomethylated Cys185 marks a predicted sugar-binding surface. Dashed lines connect to a zoom-out view showing the position of the Ca^2+^ site within the overall bent jelly-roll fold, where the locations of two putative carbohydrate-binding grooves are marked. **d** Retrospective analysis of *L. biflexa* FlaA2 cryo-EM density at the structurally equivalent Ca^2+^ binding site. Density consistent with bound calcium is observed, with coordinating residues Asp52, His102, and Asp219 labelled.

While FlaA2 structure has been determined by cryo-EM^15^, FlaA1 remained uncharacterized. The two paralogues share only ~25% sequence identity, with FlaA1 ~ 60 residues larger than FlaA2; the size difference reflects longer interspersed loops rather than insertions of discrete domains. The apparent absence of FlaA1 from *L. biflexa* filaments motivated its X-ray crystallographic analysis. Recombinant FlaA1 required iodoacetamide (IAA) and calcium for solubility; after extensive crystallization trials, the structure from *L. borgpetersenii* FlaA1 (UniProt Q04UP1) was solved at 1.82 Å resolution (Fig. 2a,b; Supplementary Table 3). FlaA1 exhibits a bent jelly-roll-like β-sandwich fold creating a deep concave groove opposite a two-faceted convex side (Fig. 2a). The concave face comprises a 5-stranded antiparallel β-sheet, while the convex side has two partly discontinuous antiparallel β-sheets (four and six strands). Fold and topology identify FlaAs as Carbohydrate Binding Modules (CBM)^21^, though not belonging to any known CBM family^22^. Crucially, a calcium cation near the domain’s triangular vertex (Fig. 2c) is octahedrally coordinated by four protein oxygens and two water molecules. The cation stabilizes two β-strands on each convex facet, explaining its role in protein stability. Re-examination of the *L. biflexa* cryo-EM map revealed density consistent with calcium at the equivalent position in FlaA2 (Fig. 2d), confirming that calcium-dependent fold stabilization is conserved across the FlaA family.

Superposition with characterized CBM structures suggests potential sugar-binding sites in FlaA1: one in its concave groove (Fig. 2c), another on a convex facet harbouring a groove with Cys185 on its floor. IAA reacted with this solvent-exposed Cys185 during crystallization, producing its stable carboxamidomethylated derivative. Within assembled filaments, FlaA2 concave crevices bind sugar moieties decorating FlaB core protomers^15^ (confirmed in this study within assembled filaments, see Supplementary Fig. 6c, d), validating the functional relevance of CBM-type recognition in core-sheath interactions. Whether FlaA1 is incorporated as a structural component within assembled filaments, possibly occupying the unfilled groove between the two FlaA2 protofilaments, remained to be determined by direct structural analysis.

### *L. interrogans* filaments exhibit a closed sheath and reveal architectural plasticity

**Fig. 3:**
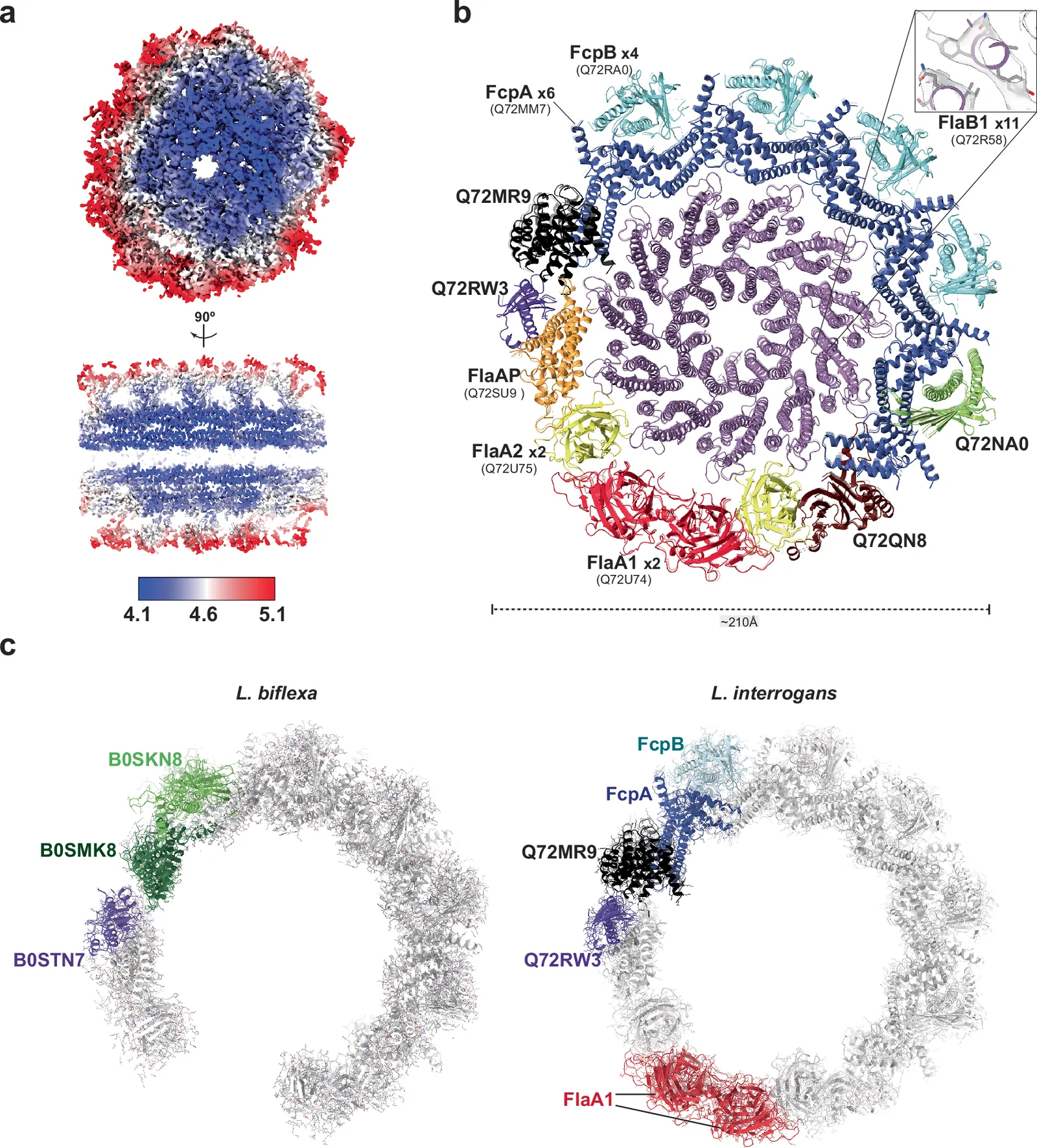
Native *Leptospira interrogans* filaments exhibit a fully closed sheath cylinder. **a** Cryo-EM map coloured by local resolution (scale bar: 4.1–5.1 Å), viewed from one end (top) and from the side (bottom), contoured at 0.04 threshold. **b** Refined atomic model shown as cartoon in transverse cross-section. Orthologous proteins are coloured and labelled as in Fig. 1 Inset: close-up of FlaB1 core density with fitted model. **c** Side-by-side comparison of lateral sheath regions in *L. biflexa* (left) and *L. interrogans* (right), highlighting species-specific differences. *L. biflexa* features B0SKN8, B0SMK8, and B0STN7; *L. interrogans* features an additional unit of FcpA and FcpB, the HEAT-repeat protein Q72MR9, Q72RW3, and FlaA1 occupying the lateral groove.

The apparent absence of FlaA1 from the *L. biflexa* cryo-EM map, together with proteomic data indicating distinct FlaB isoform profiles across species^23^, prompted us to determine the structure of flagellar filaments from pathogenic *L. interrogans*. Cryo-EM reconstruction yielded a 4.4 Å resolution map, with further density modification improving resolution to 4.2 Å (Fig. 3a; Supplementary Table 1; Supplementary Fig. 5). The atomic model was built by fitting AlphaFold-predicted orthologues into density (Fig. 3b), with *L. interrogans* proteome data^23^ and our *L. biflexa* comparative proteomics analysis guiding the assignments.

Based on differential cryo-EM features at large side chains and diagnostic glycosylation sites, the *L. interrogans* core is predominantly FlaB1, contrasting with FlaB4 in the saprophyte (Supplementary Data 3), and consistent with reported compositional analyses^20,23^. The groove on the concave face of the sheath (open in *L. biflexa*) is occupied in the pathogen by two FlaA1 protomers per sheath layer, completing the cylinder (Fig. 3b). The crystallographic monomer fitted unambiguously into this density (Supplementary Fig. 6a), resulting in a dimeric arrangement consistent with FlaA1-FlaA1 and FlaA1-FlaA2 crosslinks (Table 1). The 2-fold axis relating both monomers is perpendicular to the filament, such that the two FlaA1 protofilaments run antiparallel, each contacting distinct surfaces on their neighbouring FlaA2 partners (Supplementary Fig. 6a). Interactions with FlaA2 protofilaments open the dimer angle relative to the crystal structure (Supplementary Fig. 6b). The larger FlaA1 sugar-binding groove interfaces with FlaA2 rather than the core, while the second putative sugar-binding crevice is exposed on the filament surface, forming a continuous outward-facing groove with the dyad-related FlaA1 neighbour (Supplementary Fig. 6c). FlaA2 concave crevices bind FlaB *O*-glycans (Supplementary Fig. 6d) as in *L. biflexa*^15^. Intriguingly, the completed cylinder forms a flattened ~20 Å lateral channel bounded by FlaA1 (Supplementary Fig. 6e).

The *L. interrogans* sheath comprises 9 distinct proteins (Fig. 3b). The convex side possesses six FcpA and four FcpB units per repeat, compared to five FcpA and three FcpB in *L. biflexa*. However, the *L. biflexa*-specific protein B0SKN8 exhibits a FcpB-like fold, making the convex region compositionally similar except for the extra FcpA moiety in *L. interrogans*. The two species show the greatest architectural divergence in one sector of the sheath (Fig. 3c). In *L. interrogans*, an extra FcpA unit bridges the convex outer tier FcpB array to Q72MR9, a HEAT repeat protein^24^ that directly contacts the core (Supplementary Fig. 4e). The HEAT protein then further contacts FlaAP on the concave side, capped by the outer tier Q72RW3. In *L. biflexa*, the position of the HEAT protein is instead occupied by the mixed B0SMK8/B0SMT6 protofilament (both FlaAP paralogues). Although *L. interrogans* expresses orthologues of these FlaAP-like proteins^23^, neither was observed in its filament structure. This sector of the sheath thus represents a zone of compositional plasticity, where proteins are being differentially incorporated in each *Leptospira* species. The observed species-specific correlation between sheath organization and core compositions prompted us to investigate whether these two architectural features are coupled.

### Core flagellin identity is coupled to sheath architecture, dictating filament curvature

**Fig. 4:**
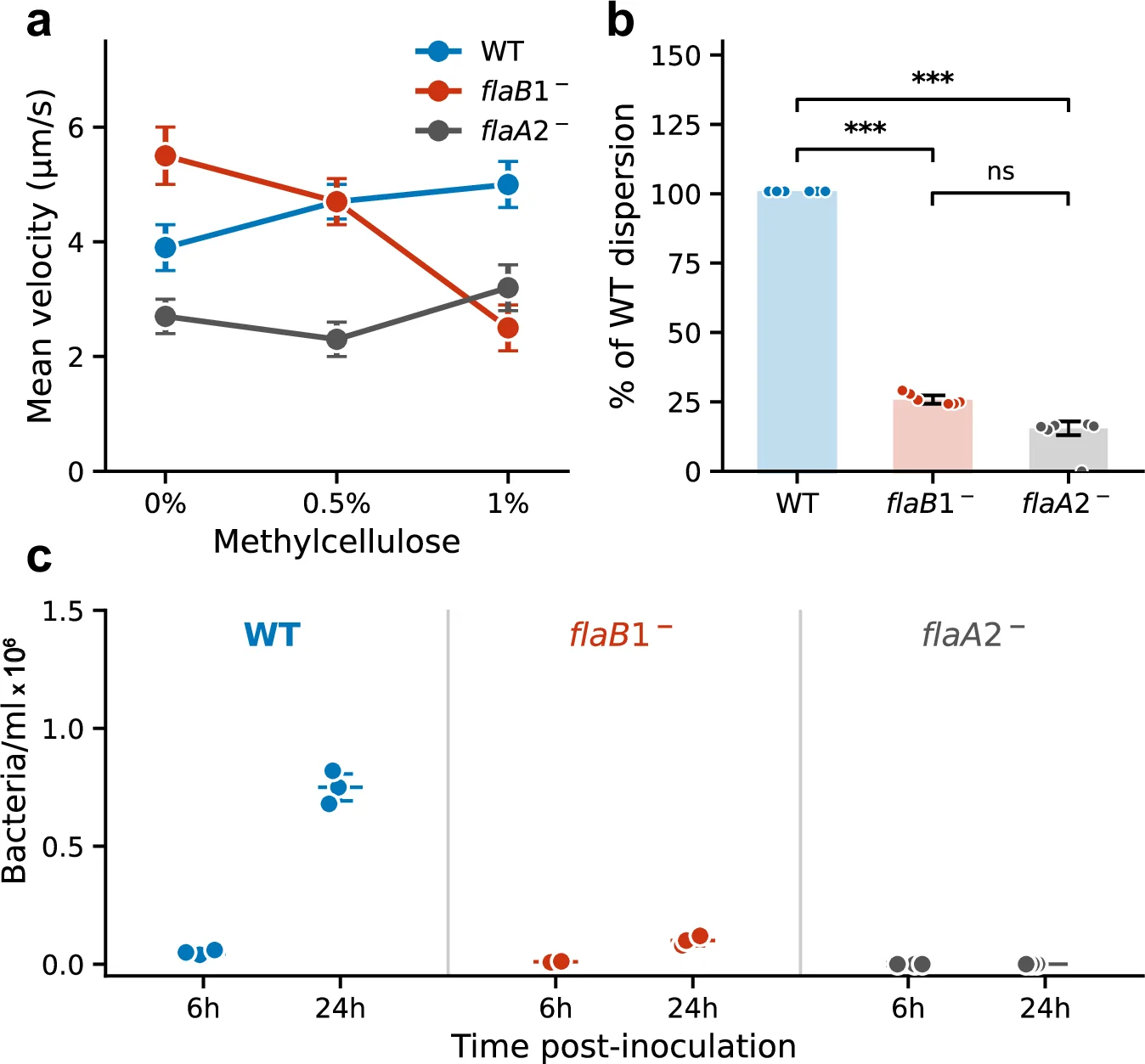
*L. interrogans flaB1*^*-*^ mutant reveals viscosity-dependent motility defects and impaired tissue invasion. **a** Swimming velocity of *L. interrogans* wild-type (WT, blue), *flaB1*^*-*^ mutant (pink), and non-motile *flaA2*^*-*^ control (grey) measured in liquid EMJH medium supplemented with increasing concentrations of methylcellulose (MC: 0%, 0.5%, 1%). Data represent mean ± s.d. from ~60 tracked bacteria per condition. **b** Dispersion in semi-solid agar (0.8%) after 10 days at 30 °C, normalized to WT. Data represent mean ± s.d. from 6 independent experiments. One-way ANOVA with Šídák’s correction (post-hoc test for multiple comparisons), resulted in *p* values: WT *vs flaA2⁻* < 0.0001; WT *vs flaB1⁻* 0.0001; and *flaA2⁻ vs flaB1⁻* 0.9946; in the plot *p* ≤ 0.0001 is marked with ***; ns = not significant. **c** Translocation across MDCK-1 epithelial monolayers in Transwell® assays. Bacteria in the lower chamber were quantified at 6 hours and 24 hours post-inoculation. The *flaB1*^*-*^ mutant shows ~8.7-fold reduction in translocation compared to WT at 24 hours, with no detectable crossing at 6 hours. Data represent mean ± s.d. from *n* = 3 independent experiments, each the mean of 3 technical replicates. Source data are provided as a Source Data file.

Extensive *O*-glycosylation of FlaB protomers on their outer surfaces dominantly mediates the native core:sheath interaction network^15^ in both species. To pinpoint whether the distinct FlaB isoforms that dominate their respective cores exert an influence on sheath organization and stability, we generated a *flaB1* deletion mutant (*flaB1*^*-*^) in *L. interrogans*. This mutant grew normally in vitro and swam indistinguishably from *L. interrogans wt* when observed by darkfield microscopy in liquid medium (Supplementary Movie 1). However, motility assays across viscosity conditions revealed striking differences (Fig. 4a). In low-viscosity medium, *L. interrogans flaB1*^*-*^ cells swam faster than *wt* (5.5 vs 3.9 µm/s), but this relationship reversed as viscosity increased: in 1% methylcellulose, mutant velocity dropped to 2.5 µm/s while *wt* increased to 5 µm/s. Consistently, the mutant showed severely impaired dispersion in semi-solid agar, dropping to 29% of *wt*, comparable to the non-motile *L. interrogans flaA2*^*-*^ control (Fig. 4b, Supplementary Fig. 7a). This viscosity-dependent motility defect translated to impaired translocation across epithelial monolayers (Fig. 4c) and, critically, complete attenuation of virulence in the hamster model of acute infection (Supplementary Fig. 7b). Thus, while the *flaB1*^*-*^ mutant assembles functional flagella, their mechanical properties are not optimized for the viscous environments encountered during tissue invasion.

**Fig. 5:**
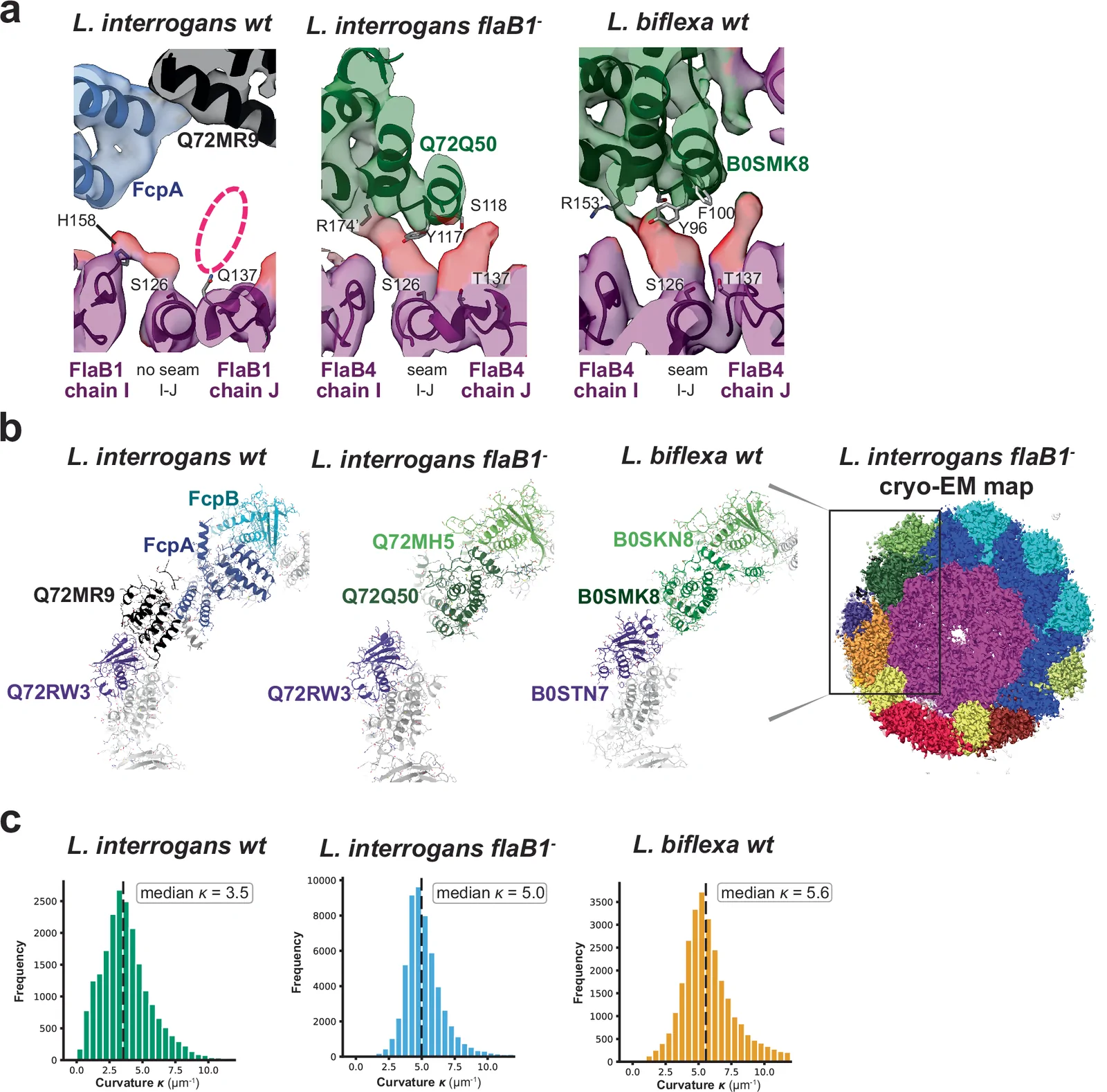
Core-driven regulation of *Leptospira* filament curvature. **a** Cryo-EM density of *L. interrogans wt* (left), *L. interrogans flaB1*^*-*^ (centre), and *L. biflexa wt* (right). Gln137 on FlaB1 (*L. interrogans wt*) cannot be glycosylated (dashed circle), creating a looser sheath interaction and no seam; Thr137 on FlaB4 (*flaB1*^*-*^ and *L. biflexa wt*) is *O*-glycosylated, likely stabilizing B0SMK8/Q72Q50 and introducing a seam within the core. The view is equivalent across the strains, focused on the I–J core protofilament interface. Cryo-EM densities are coloured according to fitted models. Glycan densities are coloured red. **b** Cryo-EM map of *L. interrogans flaB1-* (solid surface, right), coloured according to best-fitting atomic model chains (Supplementary Fig. 8c). The boxed area highlights the variable sheath region, compared across all three strains (left). The *flaB1-* sheath architecture resembles *L. biflexa* rather than parental *L. interrogans*. **c** Distribution of filament curvature (κ) for the three strains. Histograms show individual curvature measurements along filament trajectories; dashed lines indicate median values. The curvature of *flaB1-* closely matches *L. biflexa wt* rather than parental *L. interrogans wt*, correlating with sheath architectures shown above. The raw per-filament curvature measurements underlying the histograms in panel (**c**) are available at Zenodo (10.5281/zenodo.21104577).

These stark phenotypic differences prompted structural characterization of the *flaB1*^*-*^ mutant flagellar filament to identify underlying architectural bases. Cryo-EM reconstruction of *flaB1*^*-*^ filaments at 4.2 Å resolution (Supplementary Table 1; Supplementary Fig. 8) revealed that FlaB4 is indeed the most abundant FlaB isoform in the mutant core. Position 126 (serine) is glycosylated in all major FlaB isoforms. Position 137 provides a diagnostic distinction: FlaB4 carries threonine (*O*-glycosylated), whereas FlaB1 carries glutamine, which cannot be glycosylated (Supplementary Fig. 9a). The *flaB1*^*-*^map confirms FlaB4 identity through unambiguous glycan density at position 137 (Fig. 5a) and differential densities at other diagnostic side chains (Supplementary Fig. 9b). Independent quantitative proteomics of the *flaB1⁻* filaments further confirmed this assignment (Supplementary Data 3). This mutant thus provides a genetically matched *L. interrogans* background with a FlaB4-dominated core.

Remarkably, the *flaB1*^*-*^ sheath organization closely resembles that of our *L. biflexa* structure (FlaB4-dominated core) rather than that of the parental *L. interrogans* strain (Fig. 5b). The extra FcpA and HEAT repeat protein (Q72MR9) characteristic of *L. interrogans wt* are absent in the *flaB1*^*-*^ density and are replaced by *L. biflexa*-type architecture (Supplementary Fig. 9c). Consistent with this remodelling, the refined *flaB1*^*-*^ atomic model fits well into both the *flaB1*^*-*^ and *L. biflexa wt* maps, but leaves unoccupied density in the *L. interrogans wt* map where species-specific proteins reside (Fig. 5b; Supplementary Fig. 9c).

Beyond its diagnostic value, position 137 emerges as a key determinant of sheath selectivity. At the I–J protofilament interface, density consistent with the Thr137 *O*-glycan on FlaB4 lies adjacent to B0SMK8 at the core–sheath boundary (Fig. 5a); this contact is conserved in *L. biflexa wt* and *L. interrogans flaB1*^*-*^ (B0SMK8 orthologue Q72Q50). In *L. interrogans wt*, where position 137 is occupied by non-glycosylated glutamine, the conserved Ser126 glycan repositions to bridge across protofilaments I and J, eliminating the seam between them (Fig. 5a). A nearby FlaB surface loop also exhibits isoform-dependent sequence and structural differences that coincide with the altered sheath composition (Supplementary Fig. 9b). Together, distinctive glycosylation and local surface architecture of the FlaB core explain sheath protein selectivity and seam organisation.

The architectural similarities between *flaB1*^*-*^ *L. interrogans* and *L. biflexa wt* filaments also extend to their mechanical properties. Quantitative curvature analyses^25^ revealed substantial differences among the three strains (Fig. 5c). The local curvature κ (units of inverse length) is the inverse of the radius of the osculating circle that best approximates the filament’s path at each point; for example, κ = 1 µm^−^¹ corresponds to a local radius of curvature of 1 µm (Supplementary Fig. 10). *L. interrogans wt* filaments exhibited the lowest curvature (median κ = 3.5 μm^−1^; *n* = 20,977), while *L. biflexa wt* displayed significantly higher values (median κ = 5.6 μm^−1^; *n* = 28,853; difference between the two species Δκ = 2.1 μm^−1^). Critically, the *flaB1*^*-*^ mutant closely matched *L. biflexa* (median κ = 5.0 μm⁻¹; *n* = 55,579) rather than parental *L. interrogans*. This demonstrates that FlaB isoform identity, coupled with corresponding sheath architecture, primarily dictates filament curvature. The residual difference between FlaB4-containing assemblies (Δκ = 0.6 μm^−1^) likely reflects species-specific variations in minor core proteins and/or post-translational modifications.

What structural features underlie this curvature modulation? Strikingly, our structures reveal that increased curvature correlates with increasing numbers of a feature denoted a seam, where the strict helical symmetry of FlaB cores is subtly broken by a ~ 2.3 Å translational shift (Supplementary Fig. 11a). A very similar seam was observed in our previously reported structure of a *L. biflexa fcpA*^*–*^ flagellar filament, where most of the sheath was lost^15^. However, unlike the *fcpA*^*–*^ structure, which was much less curved (κ ≈ 1.3 μm^−1^) and only a single seam was observed, sheath-enclosed structures reported here contain multiple seams. *L. biflexa* (κ = 5.6 μm^−1^) exhibits four seams, whereas *L. interrogans wt* (κ = 3.5 μm^−1^) displays two (Supplementary Fig. 11b, c). This correlation between seam number and curvature aligns with longstanding models of bacterial flagellar shape determination^19,26–28^ (see below). At the same time, individual FlaB protomers display continuous conformational variation (Supplementary Fig. 11d). Thus, seams provide a structural mechanism to accommodate this flexibility while maintaining overall helical architecture.

Specific core-sheath interactions correlate with the presence or absence of seams in the *L. biflexa* and *L. interrogans wt* structures. Beyond the shared seams at interfaces K-A and B-C (each contacting a FlaA2 moiety on the sheath concave side), *L. biflexa* has two additional seams: at A-B, directly beneath the position where FlaA1 is absent, and at I-J (Fig. 5a; Supplementary Fig. 11c). B0SMK8 and FlaAP, adjacent paralogues with inverted quasi-symmetric^27^ core contacts, differ in that only B0SMK8 induces a seam. Together, these observations establish that core flagellin identity is directly coupled to sheath organization, seam distribution, and filament curvature.

## Discussion

We report high-resolution structures of endoflagellar filaments from saprophytic and pathogenic *Leptospira* species, revealing unprecedented architectural complexity in bacterial flagella. These structures comprise helically symmetric flagellin cores enveloped by 9–12 distinct sheath proteins organised along radially asymmetric architectures. Combined with genetic and phenotypic analyses, our results demonstrate that core flagellin isoform identity is coupled to sheath organization, together dictating filament curvature. Critically, this architectural plasticity proves essential for motility in viscous environments and for virulence, establishing a mechanistic framework linking protein asymmetry to the tissue-penetrating capabilities of these pathogens.

### Viscosity-dependent motility and virulence

The *L. interrogans flaB1*^*-*^ mutant provides a striking demonstration that filament architecture is tuned for specific environmental conditions. In low-viscosity liquid, the mutant swims faster than wild-type, yet this advantage reverses dramatically in viscous media, culminating in loss of dispersion in semi-solid agar and complete virulence attenuation in the mutant strain. Thus, higher-curvature FlaB4-dominated filaments appear suited to low-resistance aquatic environments, while lower-curvature FlaB1-dominated filaments are optimized for the viscous conditions encountered during tissue invasion. Flagellar architecture has similarly been linked to motility in viscous environments in externally flagellated bacteria^29^. Lower-curvature filaments are expected to generate smaller-amplitude body deformations, which may improve swimming efficiency and reduce energetic costs under high-viscosity conditions. The architectural complexity we describe here provides a structural basis for such tunability. Whether *Leptospira* actively regulates FlaB isoform expression in response to environmental cues remains unexplored.

### Mechanisms of curvature modulation

Previous work established that asymmetric sheath components promote filament curvature in *L. biflexa*: progressive loss of FcpA and FcpB leads to straightening, with wild-type filaments exhibiting curvature of ~5 µm^−1^^14^. Our structures now reveal how this curvature is achieved at atomic resolution and demonstrate an additional layer of regulation through core flagellin identity. Classical models of flagellar supercoiling proposed discrete L-state and R-state flagellin conformations mixed in varying stoichiometries^19,26,28^. However, elastic deformation within each state (quasi-equivalence) also contributes substantially to filament flexibility^27^, and recent cryo-EM structures revealed that flagellin adopts a continuum of conformations rather than two discrete states^30^. Our *Leptospira* structures are consistent with this view: FlaB protomers display continuous conformational variation, accommodated by seams where helical symmetry is locally broken^15^. The similar curvatures of FlaB4-containing *L. biflexa wt* and *L. interrogans flaB1*^*-*^ mutant filaments (median curvature difference Δκ = 0.6 μm^−1^), contrasting with the substantially lower curvature of FlaB1-dominated *L. interrogans wt* (Δκ = 2.1 μm^−1^ relative to *L. biflexa*), demonstrate that core flagellin identity, coupled with corresponding sheath architecture, primarily dictates filament curvature. The radially asymmetric sheath creates differential mass distribution around the filament axis, altering area moments of inertia that will likely generate unbalanced force vectors^31^. This is analogous to eccentric loading in elastic rods, which produces characteristic bending moments^32^. A similar principle has been demonstrated in asymmetrically decorated actin filaments where protein interactions modify bending stiffness^33^. Introducing a fully symmetric sheath (as in the *L. interrogans flaA2*^*-*^ mutant) produces straight filaments^15,34^, confirming that sheath architecture templates curvature. The asymmetric sheath architecture thus creates the mechanical context for curvature, though the detailed relationship between sheath-core contact geometry and local symmetry breaking remains to be established.

Three recent structural studies of spirochaete flagellar filaments converge with key aspects of our findings. A high-resolution cryo-EM structure of the filament from *Treponema denticola*^35^ revealed a glycosylated FlaB core encased by an asymmetric sheath of five proteins (the major FlaA, plus four minor components: FlaA1, FlaA2, FlaAP1 and FlaAP2), also demonstrating that this asymmetric core-sheath organization is critical for force transmission and motility. An independent study in *T. denticola* further confirmed several of these findings^36^. Notably, the FlaA1-FlaA2 sheath assembly in these *T. denticola* structures matches the arrangement we see in our *Leptospira* structures: an antiparallel FlaA1 dimer occupies the concave side of the filament sandwiched between two FlaA2 protofilaments. FlaA1 lacks direct contacts to the FlaB core, defining an intriguing lateral channel (Supplementary Fig. 6e) maintained across Spirochaetes.

The conserved FlaA jelly-roll fold and the asymmetric assembly principle thus extend phylogenetically from *Treponema* to *Leptospira*. However, the *Leptospira* sheath roster is substantially expanded by the additional inner and outer tier components that we describe. This elaborate sheath plausibly provides the bending stiffness required to transmit torque productively against the cell body without buckling, a particularly stringent demand in *Leptospira*. In contrast to other spirochaetes, which harbour robust bundled ribbons of overlapping filaments^37^, *Leptospira* deploys a single, comparatively short filament per cell pole that spans only a fraction of the cell length without overlap at the cell centre^10^.

In a third study, Kawamoto and colleagues describe cryo-EM 3D structures of filaments from wild-type *L. biflexa*, as well as *fcpB-* and *flaA*-family mutants^34^, resolving FcpA and FcpB sheath components that had previously been reported^14,15^. Other sheath components are missing from their maps or could not be unambiguously identified. As filament purification requires careful optimization to retain the broader sheath roster^15^, we attribute these differences to sample preparation rather than biology. Without the broader sheath context, the Kawamoto study could not address how filament curvature is biologically modulated. By comparing saprophytic and pathogenic *Leptospira*, our work identifies core flagellin isoform identity as the upstream regulatory feature selecting among functional sheath configurations, with the *flaB1*^*-*^ virulence-attenuation phenotype demonstrating its biological consequence.

### Core-sheath coupling

Our data reveal a hierarchical, unidirectional relationship in which core identity determines sheath architecture, which in turn dictates curvature. Perturbing the sheath does not alter core composition, as shown by *flaA2*^*-*^ mutants in both *L. interrogans*^15^ and *L. biflexa*^34^. These mutants assemble symmetric sheaths yet retain their native FlaB isoform composition, producing straight, non-motile filaments. In contrast, perturbing the core reorganizes the sheath. The *L. interrogans flaB1*^*-*^ mutant, in which FlaB4 replaces FlaB1 as the dominant core flagellin (Supplementary Data 3), assembles a *L. biflexa*-like sheath architecture and exhibits correspondingly increased curvature (Fig. 5), while remaining functional and tuned for different mechanical requirements (Fig. 4). Thus, core identity acts upstream, selecting among functional sheath configurations, while loss of sheath asymmetry collapses the system outside its physiological operating range.

What molecular features enable this selectivity? Position 137 provides a direct structural answer (Fig. 5a): the Thr137 *O*-glycan on FlaB4 stabilizes B0SMK8 at the I–J interface, whereas Gln137 on FlaB1 cannot be glycosylated, abolishing this contact and triggering Ser126 glycan rearrangement that eliminates the I–J seam. Electrostatic complementarity reinforces this selectivity: the FlaB1 core exposes a more cationic surface than FlaB4, particularly at loop 142-RGSR-145, further disfavouring B0SMK8 binding while permitting FcpA substitution.

### Conservation beyond *Leptospira*

Comparative structural analysis revealed clusters of structurally similar sheath proteins within species (orthologue variants) and across Spirochaete genera (paralogues), despite sequence divergence (Supplementary Table 4). Only some of the proteins from each cluster were observed in our cryo-EM structures, even though their paralogues were clearly detected by LC-MS/MS (Supplementary Data 2). This raises the possibility of compositional plasticity within the sheath, the nature of which remains to be characterised. One attractive scenario, awaiting direct experimental testing, is that variant components are differentially incorporated at distinct lifecycle stages or at specific filament locations, providing a means of dynamically modulating filament architecture and, by extension, swimming behaviour, in response to environmental cues.

FlaA proteins are conserved phylum-wide, often as multiple paralogues, with *Treponema* and *Brachyspira* also possessing FlaA2-binding proteins, suggesting conserved roles in asymmetric sheath assembly. In contrast, FcpA and FcpB are unique to *Leptospira*^10,12,13^, representing genus-specific adaptations for enhanced torque transmission through a single filament per cell pole.

Spirochetal FlaA proteins define a new carbohydrate-binding module family characterized by a bent jelly-roll topology, calcium-dependent fold stabilization, and conserved sugar-recognition grooves. FlaA2 binding to FlaB *O*-glycans demonstrates functional CBM activity^15^. Intriguingly, FlaA1 exposes a putative sugar-binding groove on the filament surface, with consecutive dimers spaced ~50 Å apart, within the range of peptidoglycan oligosaccharide spacing^38^, suggesting possible filament–cell wall interactions.

Finally, we note that the presence of a lateral channel adjacent to the FlaA1 dimer may also be relevant to dynamic filament shape switching. *Leptospira* can reverse direction very rapidly, implying filament supercoil handedness transitions on the millisecond timescale^39^, which must be accommodated by seam transitions^19,26–28^ and/or other substantial core rearrangements^30^. However, core-sheath interactions reported here and in ref. ^15^, appear highly selective for a given seam configuration, indicating that the sheath locks the seams into place. How, then, is this architecture compatible with rapid shape transitions? The timescale appears far too short to permit sheath compositional rearrangement. Instead, we speculate that flagellar seam transitions localize to the FlaA1 lateral channel, where the absence of direct core-sheath contacts and the apparent flexibility of FlaA1–FlaA1 interfaces (Supplementary Fig. 6b) may allow seam remodelling by relieving the local mechanical constraints that elsewhere lock the sheath onto the core. Seam variability in this region of our structures (compare Supplementary Fig. 11b, c) supports this interpretation, as does the finding that *Leptospira flaA1*^*-*^ deletion strains maintain motility and infectivity^9^. Moreover, the observation of a nearly identical FlaA1 lateral channel in *Treponema*^35,36^ suggests that this mechanism may generalize to other sheathed flagellar filaments.

### Conclusions

This study introduces the most complex flagellar filament architecture characterized to date in bacteria, revealing how protein asymmetry creates mechanically tuned assemblies. The demonstration that core flagellin identity is coupled to sheath organization, together dictating curvature, provides a structural basis for environment-specific motility. How the asymmetric filament couples mechanically to the crosslinked spirochetal hook during torque transmission remains an open question. The complete virulence attenuation of the *flaB1*^*-*^ mutant establishes that architectural tunability is essential for tissue invasion by pathogenic *Leptospira*, and may represent a conserved strategy enabling spirochaetes to optimize motility across the diverse environments encountered during their complex lifecycles.

## Methods

### Bacterial strains and culture conditions

*Leptospira biflexa* serovar Patoc strain Patoc I wild-type (*wt*; reference genome NCBI BioProject PRJNA20133^40^), *fcpA*^*-*^ knockout mutant^10^, and *flaA2*^*-*^ (this study). *L. interrogans* serovar Manilae strain UP-MMC-NIID LP *wt* and *flaA2⁻*^9^, and *flaB1⁻* (this study). As reference for *L. interrogans* protein identifiers, the thoroughly annotated WGS of *L. interrogans* serovar Copenhageni strain Fiocruz L1-130 was used^20,41^; the loci referenced here are highly conserved across *L. interrogans* serovars. All strains were cultured in liquid Ellinghausen-McCullough-Johnson-Harris (EMJH) medium at 30 °C^42^. Cultures were grown to mid-logarithmic phase (optical density ~0.3 at 420 nm) before harvest for flagellar purification.

### Generation of *Leptospira* mutant strains

The *L. interrogans* serovar Manilae *flaB1*^*-*^ mutant (disruption of gene LIMLP_09410) was obtained by transposon mutagenesis using Himar1 as previously described^43^. The transposon insertion site was confirmed by PCR with synthetic primers flaB1F (5’-TATCTAAGAAGCTGCAGAAC-3’) and flaB1R (5’-GATCATTAATCACAACTTAG-3’) flanking the transposon insertion sites. Whole-genome sequencing was also performed to rule out off-target modifications. Briefly, genomic DNA from *L. interrogans* serovar Manilae wild-type (WT) and *flaB1*^*–*^ strains was extracted with the Maxwell 16 cell DNA purification kit (Promega) and sequenced by Illumina next-generation sequencing. Sequence Reads were cleaned using fqCleaner and mapped to the reference genome of *Leptospira interrogans* serovar Manilae strain UP-MMC-NIID LP (accession numbers CP011931, CP011932, CP011933) using the Burrows-Wheeler Alignment tool version 0.7.5a. The detection of single nucleotide polymorphisms (SNPs) and insertions or deletions (Indels) was performed utilizing the Genome Analysis Toolkit (GATK2), adhering to the recommended practices by the Broad Institute. For targeted mutagenesis of *flaA2* in *Leptospira biflexa*, a suicide vector carrying a kanamycin resistance cassette flanked by 1 kb of the *flaA2* flanking sequences was synthesized by GeneArt (Life Technologies, Grand Island, NY, USA), pretreated by UV, and used to transform *L. biflexa* as previously described^44^. Cells were selected on EMJH agar containing 50 μg/mL kanamycin, and double-crossover mutants were identified by PCR.

### Motility assays

(i). Dispersion in semi-solid medium: mid-logarithmic phase *Leptospira* cultures (approximately 2×10⁸ bacteria/mL) were inoculated (3 µL) into 0.8% soft-agar medium supplemented with EMJH and incubated for 10 days at 30 °C. Six dispersion halo diameters were measured manually and normalized to *wt* values. *L. interrogans flaA2*^*-*^ was used as a non-motile control. Experiments were performed in at least three independent replicates per strain.
(ii). Velocity measurements in varying viscosity: late-logarithmic phase cultures were diluted in fresh EMJH medium with or without methylcellulose (0%, 0.5%, or 1% from a 2% stock solution in water) to obtain approximately 20 bacteria per field of view. A 5 µL droplet of diluted culture was deposited on a glass slide and covered with a coverslip elevated by silicone spots to minimize compression against the glass surface and Brownian motion. Slides were observed using an Olympus BX53 darkfield microscope connected to a Hamamatsu Orca Flash camera. Multiple 20-second videos were recorded using CellSens Dimension software (Olympus). Approximately 60 bacteria per strain were manually tracked to determine mean swimming velocity.

Statistical analyses and data plotting were performed using GraphPad Prism 9.5.0.

### Epithelial translocation assay

The ability of *Leptospira* strains to cross epithelial barriers was assessed using a Transwell® assay as previously described^45^. Briefly, canine kidney epithelial cells (MDCK-1) were seeded onto Transwell® cell culture inserts (3.0 µm porosity) and cultured at 37°C with 5% CO₂ in Eagle’s Minimum Essential Medium (EMEM) supplemented with 10% inactivated foetal bovine serum and 2 mM L-glutamine until confluent. Cell layer integrity was verified using trypan blue exclusion. Inserts were washed with an EMJH/EMEM mixture (1:2 ratio), and mid-logarithmic *Leptospira* (4×10⁷ bacteria/mL) were added to upper chambers. Bacteria that successfully crossed the cell layer were collected from lower chambers and counted manually using a Petroff-Hausser chamber at 6 hours and 24 hours post-inoculation. Generation time of *L. interrogans* (~20 hours) was considered for bacterial load calculations at the 24 hours timepoint. Statistical analyses and data plotting were performed using GraphPad Prism 9.5.0.

### Animal infection model

Four-week-old Golden Syrian hamsters (RjHan:AURA, Janvier Labs; *n* = 4 per group; all males to reduce variability) were infected by intraperitoneal injection with 10⁶ *Leptospira interrogans* serovar Manilae (*wt* or *flaB1*^*-*^) counted with a Petroff-Hausser counting chamber. Animals were monitored daily and euthanized by CO_2_ inhalation upon reaching predefined endpoint criteria (signs of distress and morbidity). Survival was recorded for 20 days post-infection. All animal procedures were approved by the Institut Pasteur Animal Care and Use Committee (CETEA #220016) and the French Ministry of Agriculture, in accordance with European Union Directive 2010/63/EU for the protection of animals used for scientific purposes.

### Flagellar filaments purification

Flagellar filaments from *L. biflexa* and *L. interrogans* strains were purified using reported protocols^10^. Purified filaments were assessed by SDS-PAGE and negative-stain transmission electron microscopy to confirm purity and structural integrity.

### Single particle analysis cryo-electron microscopy

(i). Sample preparation: purified flagellar filaments at ~0.5–1.0 mg/mL were applied to Quantifoil R1.2/1.3 Cu300 mesh holey carbon grids; prior to sample application, all grids were plasma discharged in a Gatan Model 950 Solarus Advanced Plasma System using H_2_O_2_. Grids were blotted for 6 seconds, with the blotting force parameter set to 2, at 18 - 22 °C and 100% humidity using a Vitrobot Mark IV® (Thermo Fisher Scientific) before plunging into liquid ethane.
(ii). Data collection: cryo-EM data were collected on a Titan Krios operated at 300 kV equipped with a Quantum LS Model 1967 energy filter (20 eV slit width) and a K3 direct electron detector. Micrographs were recorded in super-resolution counting mode at nominal magnification of 81,000× (calibrated pixel size of 1.068 Å for all samples, with a super-resolution pixel size 0.534 Å) using Serial-EM^46^. Total electron doses of ~25 and ~60 e⁻/Å², for *L. biflexa* and *L. interrogans* samples, respectively, were fractionated across 42 frames with a frame time of ~0.1 s. Defocus values ranged from −1.5 to −2.6 μm. Total dataset sizes: 1985, 1081, 1094 micrographs for *L. biflexa wt*, *L. interrogans wt* and *L. interrogans flaB1*^*-*^, respectively.
(iii). Image processing: cryo-EM refinement and 3D reconstruction were performed using cryoSPARC^16^ using a specialized workflow to account for supercoil deviations from strict helical symmetry. CryoSPARC version 3.3.2 was used for all except the final stages of refinement, which used the updated version 4.6.2 where noted below. All three structure refinements followed the same general protocol (Supplementary Figs. 2, 5 & 8a). After initial micrograph processing (full-frame motion correction, patch CTF estimation), the filament tracer tool was first applied for particle picking using the template-free option, followed by one or more rounds of 2D classification and manual particle selection. The filament tracer tool was then run a second time using the final manually selected 2D classes as templates. Particles identified by this latter step were initially extracted with 192 × 192 box size and 2.136 Å nominal pixel size, followed by multiple rounds of 2D classification and manual selection to eliminate false-positive junk particles such as high-contrast carbon edges. The resulting particles were subjected one or more times to non-symmetrized helical refinement (option ‘maximum symmetry order to apply during reconstruction’ set to 1), starting with a generic sheath-less flagellar filament reference volume filtered to 20 Å resolution, resulting in reported resolutions of ~6 Å. The resulting refined particle alignments were then used to re-extract 384 box-size, 1.068 Å nominal pixel-size image segments. Following additional rounds of helical refinement, 2D classification, local/global CTF refinement and/or anisotropic magnification correction, the heterogeneous refinement job was used to generate 3-5 structure classes. Particles belonging to artefactual volumes or structures with missing sheath densities were then discarded. Classes that were kept included volumes that were rotated and translated with respect to each other by the bacterial flagellar pseudo-symmetry operator (+/- ~ 26 Å z-shift and +/- ~ 32.7° z-rotation). The final stage of structure refinement utilized the ‘non-uniform refinement’ job, which overcame local minima due to pseudo-symmetric false alignments but also shifted some nearby particles on top of each other. This latter effect introduced particle duplications between the two gold standard half data sets, artificially boosting FSC resolution estimates. This resolution artefact was subsequently eliminated by running a ‘remove duplicates’ job, followed by a ‘local refinement’ job where additional particle duplications were prevented by restricting particle translation and rotation changes to +/- 7.5 Å (15 Å extent) and +/- 5° (10°), respectively (recentring of rotations and shifts after each iteration was disabled). Reported final map resolutions correspond to the final, auto-tightened mask generated by cryoSPARC. Density modification was subsequently performed using RESOLVE^17^ from the Phenix package^47^ to improve map interpretability for structure building. This required removing low-frequency background solvent fluctuations from 3D map corners, which was accomplished by using relion_image_handler^48^ to extract the central 256^3^ zone from final cryoSPARC output raw 384^3^ half-maps. An estimated protein occupancy of 0.52451 was input to RESOLVE based on identified protein components, flagellar geometry, and volume size. Final map gold-standard FSC resolutions were 3.5 Å (176,558 particles) for *L. biflexa*, 4.4 Å (35,760 particles) for *L. interrogans wt*, and 4.3 Å (53,501 particles) for *L. interrogans flaB1*^*-*^ mutant (Supplementary Fig. 12a). Estimated resolution improvements reported by RESOLVE were ~0.15-0.2 Å. The distribution of particle viewing directions was as expected for elongated filaments (Supplementary Fig. 12b). Side views predominated, yet with a full range of in-plane (azimuthal) orientations well populated, and end-on views sparsely sampled. Local resolution was calculated using the Phenix package (phenix.local resolution)^47^.
(iv). Model building: FlaB models were predicted with AlphaFold3^49^, rigid-body fitted into the cryo-EM maps with ChimeraX v1.10.1^50^, and initially refined interactively using Coot v1.1.18^51^. Sheath proteins were initially fitted within cryo-EM maps similarly, using: (i) crystal structures of FcpA and FcpB^14^, including required sequence substitutions for each *Leptospira* species; (ii) AlphaFold3 predictions for remaining components; and (iii) crosslinking mass spectrometry data to decide in ambiguous cases. For the *L. interrogans flaB1*^*-*^ mutant, initial sheath protein models were derived from the *L. biflexa* structure given the architectural similarity revealed by cross-correlation analysis (see Results), with sequence substitutions for *L. interrogans* orthologs where applicable. Models were further refined interactively with Coot using intra-chain self-restraints (6 Å sphere), and real-space chain refinement with Geman-McClure restraints (α parameter 0.01). A conservative approach was followed for all structures with backrub rotamers, deleting side chains, residues or short loops in regions where the cryo-EM density was not reliable (undetectable density at sensible contour levels). Model validation was performed continuously within Coot. Secondary structure restraints were calculated from the starting models using phenix.secondary_structure_restraints as implemented in Phenix v1.21.2-5419^47^. These extra restraints were included in the first real-space refinement cycles with phenix.real_space_refine v2.0-5936 using Ramachandran restraints^52^. Servalcat v0.4.128^53^ was then used to further refine atomic models in reciprocal space against the final unsharpened and unweighted cryo-EM half-maps. External geometric restraints based on initial structures (ProSMART v0.860^54^) were used throughout Servalcat refinement. Final validation was done with MolProbity^55^ implemented within Phenix. Checkmysequence v1.5.3 was instrumental to validate sequence assignment and correct map interpretation errors^56^.

### Quantitative filament curvature analyses

Helical filament coordinates were extracted from RELION star files following helical refinement using custom bash scripts (Supplementary Software 1 & 2). Particle positions were corrected for origin shifts by subtracting rlnOriginXAngst and rlnOriginYAngst values from micrograph coordinates (in pixels) multiplied by pixel size (0.534 Å for all three data sets), yielding actual 2D trajectory positions in Ångstroms. Three-dimensional coordinates were reconstructed from 2D positions and tilt angles (rlnAngleTilt) by iteratively calculating z-coordinates as z(*n*) = z(n-1) + cos[(θ(n) + θ(n-1))/2] × d(n,n-1), where d represents the Euclidean distance between consecutive particles in the xy-plane.

Local curvature (κ) and torsion (τ) were computed using the method of Crenshaw^25^ with a separation parameter of 10 particles. For each filament segment spanning 2×sep positions, the algorithm constructs tangent (T), normal (N), and binormal (B) vectors to determine local geometric properties. Curvature κ* at position i is calculated as κ*(i) = 2·arctan(sin φ/cos φ)/(|C|+ |D|), where φ is the angle between vectors C and D spanning the segment, and the final curvature is the average of consecutive κ* values multiplied by 10^4^ for convenient scaling. Torsion τ is calculated as the angle between consecutive binormal vectors along the trajectory.

Individual curvature measurements were treated as sampling points of the intrinsic filament curvature, with random perturbations introduced during cryo-preservation averaged out across high n. *L. biflexa wt* (median κ = 5.6 μm^−1^, interquartile range IQR 4.5–7.1; *n* = 28,853 from 409 filaments), *L. interrogans flaB1⁻* (median κ = 5.0 μm^−1^, IQR 4.3 - 6.0; *n* = 55,579 from 1148 filaments), and *L. interrogans wt* (median κ = 3.5 μm^−^^1^, IQR 2.5–4.8; *n* = 20,977 from 309 filaments), were compared using non-parametric methods. Overall differences were assessed using the Kruskal-Wallis test (H = 15,798, *p* « 0.0001); pairwise comparisons used Mann-Whitney U tests (all *p* « 0.0001). Given the large sample sizes, effect sizes provide more informative measures of difference magnitude than *p*-values: Cohen’s d was 0.83 (large) for *L. biflexa vs L. interrogans wt*, 0.60 (medium) for *flaB1*^*-*^ *vs L. interrogans wt*, and 0.25 (small) for *L. biflexa vs flaB1*^*-*^, indicating that the FlaB4-dominated strains (*L. biflexa wt* and *L. interrogans flaB1*^*-*^) cluster together while FlaB1-dominated *L. interrogans wt* is set apart. Bootstrap 95% confidence intervals for median differences confirmed non-overlapping intervals for all comparisons (Δκ = 2.02 [1.98–2.05], 1.44 [1.41–1.47], and 0.58 [0.55–0.60] μm^−1^, respectively). All statistical analyses were performed in Python 3.12 using SciPy and NumPy.

### Comprehensive mass spectrometry and crosslinking network analyses

(i). Label-free quantitative proteomics of *L. biflexa* filaments: purified flagellar filaments from *L. biflexa wild-type*, *fcpA*^*-*^ and *flaA2*^*-*^ strains (~20 µg; three replicates for each) were embedded into polyacrylamide gels by short electrophoresis in the presence of SDS, and Coomassie-stained to excise the protein band. Gels were destained, reduced with dithiothreitol, alkylated with iodoacetamide, and digested overnight with sequencing-grade trypsin. (Promega) as described previously^57^. Peptides were extracted with 60% acetonitrile, 0.1% trifluoroacetic acid, dried, desalted with ZipTip® C18 tips (Millipore) and resuspended in 0.1% formic acid. Samples were analysed by LC–MS/MS on an UltiMate 3000 nano-HPLC (Thermo Fisher Scientific) coupled to a Q-Exactive Plus mass spectrometer (Thermo Fisher Scientific). Peptide separation was performed on an Easy-Spray analytical column (PepMapTM RSLC, C18, 75 µm X 50 cm, 2 µm particle size), using a 90-min 1–35% acetonitrile separation gradient at 200 nL.min^−1^ (mobile phases: 0.1% formic acid in water, 0.1% formic acid in acetonitrile). Mass analysis was carried out in a data-dependent mode: acquisition of full MS scans in a range of m/z from 200 to 2000; followed by HCD fragmentation of the 12 most intense ions in each segment, using a stepped normalized collision energy of 25, 30, and 35 and a dynamic exclusion list. Protein identification and label-free quantification were performed with PatternLab for Proteomics V^58^. A target-reverse *L. biflexa* serovar Patoc database (downloaded from UniProt in 12/2021) was used for protein identification. Oxidation of methionine was included as a variable, and carbamidomethylation as a fixed modification. Initial mass tolerance for the measured precursor m/z was 35 ppm. Peptide spectrum matches were filtered to achieve <10 ppm of tolerance for the precursor, and less than 1% of false discovery rate (FDR) at the protein level. Differential abundance analyses separately compared *wild-type L. biflexa* with *fcpA*^*-*^ and with *flaA2*^*-*^ mutants using spectrum counting. PatternLab V was used to statistically identify proteins exclusively detected in each condition (*p* < 0.05) and to pinpoint proteins with an FC > 2 (*q* < 0.05).
(ii). Label-free quantitative proteomics of *L. interrogans* filaments: purified flagellar filaments from *L. interrogans flaB1⁻* strain (five biological replicates) were diluted in 8 M urea, 100 mM Tris-HCl pH 8.5, reduced with 5 mM TCEP, alkylated with 10 mM iodoacetamide and digested in solution sequentially with LysC (Promega; 4 h at 30 °C) and trypsin (Promega; overnight at 37 °C, after dilution to <2 M urea). Peptides were desalted on Sep-Pak C18 cartridges (Waters), eluted in 50% acetonitrile/0.1% formic acid, dried, and resuspended in 2% acetonitrile/0.1% formic acid. LC-MS/MS analysis was performed on a Q-Exactive Plus mass spectrometer (Thermo Fisher Scientific) coupled to a Proxeon EASY-nLC 1200, using a 40 cm home-made C18 column (ReproSil-Pur Basic C18, 1.9 µm, 100 Å; Dr. Maisch) heated to 60 °C, with a 165-min multi-step acetonitrile gradient at 250 nL/min. Survey MS scans were acquired at 70,000 resolution and MS/MS at 17,500 (top-10 data-dependent acquisition, 45 s dynamic exclusion, isolation window 1.6 m/z, HCD NCE 28). Raw files were processed with MaxQuant v2.8.0.0 (Andromeda search engine) against the *L. interrogans* serovar Manilae proteome (PATRIC 214675.5, 4,561 entries). Trypsin specificity was set with up to two missed cleavages. Methionine oxidation and protein N-terminal acetylation were included as variable modifications, and carbamidomethylation as a fixed modification. Precursor and fragment mass tolerances were 4.5 ppm and 20 ppm, respectively. Match between runs was enabled (match time window 0.4 min, alignment time window 20 min). One unique peptide was required per protein group, and both peptide and protein FDR were set to 1%. FlaB-isoform relative abundances were derived from the resulting label-free intensities.
(iii). Crosslinking mass spectrometry: purified *Leptospira* filaments were treated with NNP9 following established protocols^59^, including some adaptations. Briefly, NNP9 was used at 5 nmol per 10 µg protein, for 1 hour at 4°C to capture native protein: protein contacts. Reactions were quenched with ammonium bicarbonate, and crosslinked complexes were purified by enhanced filter-aided sample preparation (eFASP) on photocleavable agarose beads. After on-filter tryptic digestion, peptides were released by UV irradiation (365 nm), dried, and resuspended for LC–MS/MS analysis on a Q-Exactive HF mass spectrometer (Thermo Fisher Scientific). Crosslinked peptides were separated on a home-made C18 nanocolumn (75 µm ID, solid phase: Aeris C18, 1.7 µm, Phenomenex) using a 90 min hydrophobic gradient in the presence of 0.1% formic acid (from 8% to 30% B [80% acetonitrile, 1% formic acid]) in 70 min, followed by a step up to 60% B in 20 minutes). Data-dependent acquisition was performed in positive polarity at 60,000 resolution (both MS and MS/MS) from 300 to 1800 m/z in MS and 200 to 2000 m/z in MS/MS, excluding singly and doubly charged species. Crosslinks were identified using Mass Spec Studio^60^ with ±10 ppm mass tolerance and filtered to 1% FDR after removal of peptides identified in less than 2 MS/MS events. Crosslink spectrum matches were then carefully checked manually. Validated crosslinked residue pairs were mapped onto structural models using PyMOL/PyXlinkViewer, and interaction networks were visualized in xiVIEW^61^ (https://www.xiview.org), enabling integrative interpretation of filament architecture and inter-subunit connectivity. The crosslinking interaction network (Fig. 1c) was exported for interactive web-based exploration, publicly accessible at https://www.xiview.org/network.php?upload=42923-71803-52415-45391-89719; allowing users to explore individual crosslinked residue pairs, filter by confidence scores, and examine protein-protein connectivity.

### FlaA1 X-ray crystallographic analysis

(i). FlaA1 expression and purification: *L. borgpetersenii* FlaA1 (UniProt Q04UP1) was expressed in plasmid pET32a^62^ from a truncated construct (lacking the first 49 and last 16 residues) with a TEV-cleavable N-terminal 6xHisTag, in *E. coli* Lemo21 cells cultured in TB medium. The protein was overexpressed for 18 hours at 20 °C with 1 mM IPTG in the presence of ampicillin (100 μg/mL) and chloramphenicol (34 μg/mL) under agitation (220 rpm). FlaA1 was purified in the presence of 10 mM CaCl_2_ by standard nickel affinity chromatography (HisTrap FF, Cytiva), TEV digestion and size exclusion chromatography (Superdex S75 10/300, Cytiva). Purified protein was treated 1 hour with 10 mM dithiothreitol, followed by 1 h with 55 mM iodoacetamide at RT.
(ii). Crystallization and structure determination: robotic screening using commercial sparse-matrix kits led to several hits. Optimized crystals using hanging-drop vapour diffusion grew in 0.1 M sodium cacodylate pH 6.5, 1 M ammonium sulphate, with 2 μL protein (50 mg/mL) mixed with 2 μL reservoir solution. Crystals were cryoprotected in reservoir solution supplemented with 25% (v/v) glycerol before flash-freezing in liquid nitrogen. Diffraction data were collected at Diamond Light Source beamline I04 at 100 K. Data were processed using XDS^63^ and scaled with Aimless^64^. The structure was solved by molecular replacement using Phaser^65^ with an AlphaFold3-predicted model as search probe. The structure was interactively rebuilt using Coot 1.1.18 and refined with phenix.refine 1.21.2-5419^66^ to 1.82 Å resolution in space group P2_1_2_1_2_1_. Data processing and model refinement statistics are detailed in Supplementary Table 3. Coordinates and structure factors have been deposited in the PDB (accession code pdb_00009cyn).

### Comprehensive structural homology analysis and cross-spirochaete comparison

Structural homology searches were carried out with FoldSeek^67^ installed locally, using all known *Leptospira* filament protein structures as queries against: (i) entire proteomes from *L. biflexa* (NCBI Taxonomy Identifier 456481) and *L. interrogans* (taxid 267671) as targets to identify paralogues, fetched from UniProt and associated AlphaFold Database entries; and, (ii) *Treponema pallidum* (taxid 243276), *Borrelia burgdorferi* (taxid 224326), and *Brachyspira hyodysenteriae* (taxid 1266923) entire proteomes, to identify spirochetal orthologues. Predicted structures from entire proteomes were fetched using a script (Supplementary Software 3). FoldSeek “easy-search” routine was used with default search parameters, except for not prefiltering (exhaustive-search); using an automatically optimized number of iterations; and including average LDDT and probability prob in the output table (to better identify domain similarities and not only entire protein fold similarities). A specific E-value threshold was not used; a contrast line between hits and noise was preferred instead. Structural families resulted in ≥0.7 estimated probability for query and target to be homologous. Sequence identities within families were calculated using clustalW^68^. Protein copy numbers for *L. interrogans* were extracted from quantitative proteomic datasets^23^ if available.

### Reporting summary

Further information on research design is available in the Nature Portfolio Reporting Summary linked to this article.

## Supplementary information

Supplementary Information

Description of Additional Supplementary Files

Supplementary Data 1

Supplementary Data 2

Supplementary Data 3

Supplementary Movie 1

Supplementary Software 1-3

Reporting Summary

Transparent Peer Review file

## Source data

Source Data

## Data Availability

Atomic coordinates of filament models and corresponding cryo-EM maps have been deposited in the worldwide Protein Data Bank and Electron Microscopy Data Bank under accession codes pdb_000010LM/EMD-75270 for *L. biflexa wt*; pdb_000010LK/EMD-75268 for *L. interrogans wt*; and pdb_000010LL/EMD-75269 for *L. interrogans flaB1*^*-*^. Atomic coordinates and X-ray diffraction structure factors for the FlaA1 crystal structure have been deposited in the wwPDB under accession code pdb_00009cyn [https://www.rcsb.org/structure/9CYN]. The mass spectrometry proteomics data have been deposited to the ProteomeXchange Consortium via the PRIDE^69^ partner repository with the dataset identifiers PXD072058 for the *L. biflexa wt* and *flaA2*^*-*^ strains; PXD030741 for the *L. biflexa fcpA*^*-*^ strain^15^; PXD077813 for the *L. interrogans flaB1*^*-*^ strain; and, PXD073467 for the crosslinking data. Whole-genome sequencing data of the generated *Leptospira* mutant strains are available at NCBI under BioProject accession # PRJNA1416034. Source data are provided with this paper. The filament curvature raw data for the three strains studied in this work are available at Zenodo under 10.5281/zenodo.21104577. Source data are provided with this paper.

## Code availability

The custom scripts used for filament coordinate extraction (Supplementary Software 1), curvature analysis (Supplementary Software 2) and spirochaete proteome retrieval (Supplementary Software 3) are available at Zenodo under 10.5281/zenodo.21104577.
