# Supplementary material for: Core–sheath coupling controls flagellar curvature and motility in Leptospira
Source: Nat Commun. 2026 Jul 25;17:9075. doi: 10.1038/s41467-026-75995-6 (PMC13518957; doi:10.1038/s41467-026-75995-6)
Supplement: Supplementary file 1 [file 41467_2026_75995_MOESM1_ESM.pdf]

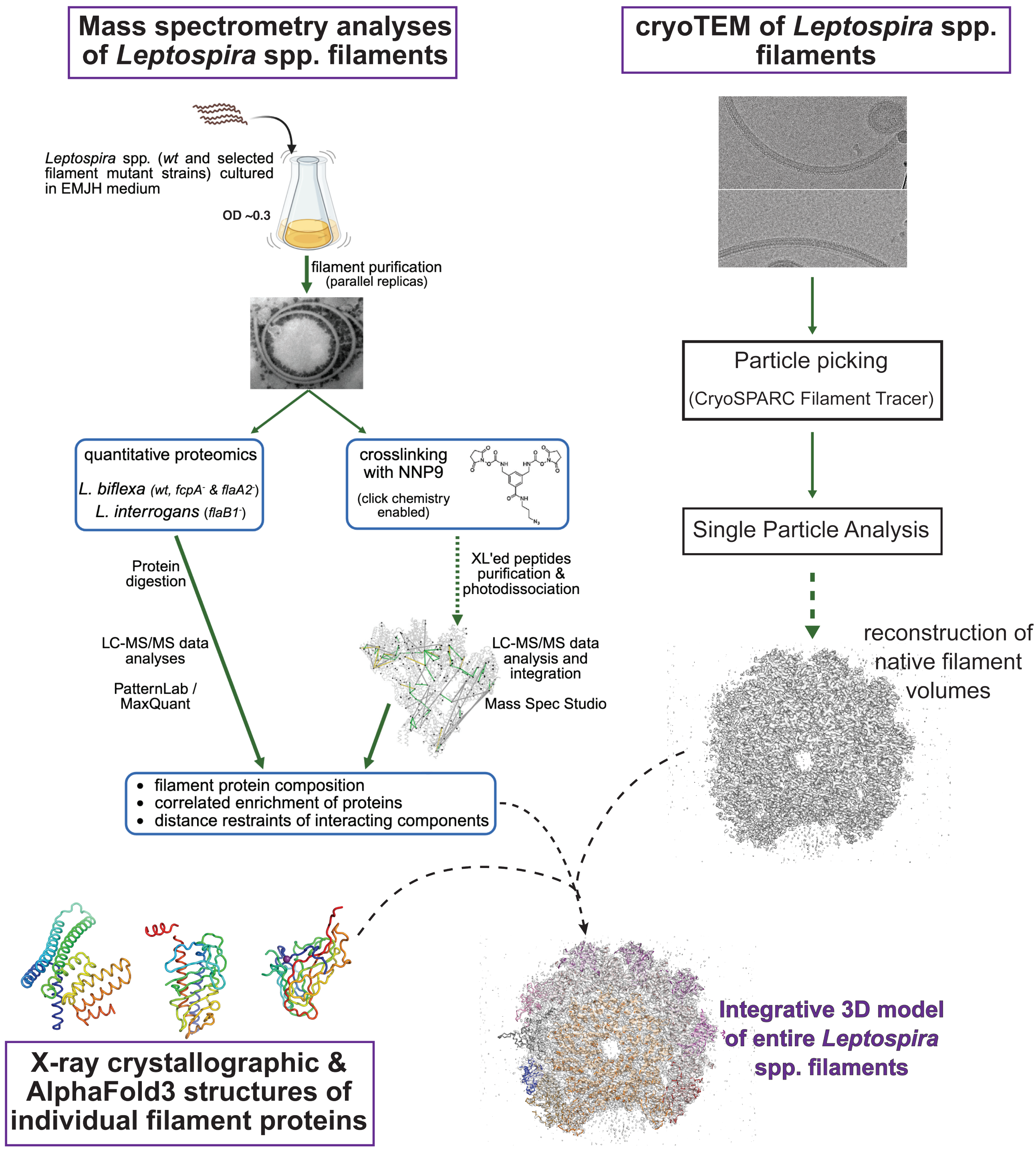

Supplementary Fig. 1. Integrative structural biology workflow. Schematic overview of the integrative approach combining three methodological branches: (i) cryo-EM single particle analysis of purified filaments to obtain 3D reconstructions (right); (ii) mass spectrometry, including comparative proteomics of *wt vs* sheath mutants (*flaA2*-, *fcpA*-) and crosslinking MS to derive distance restraints and identify co-enriched components (left); (iii) X-ray crystallographic and AlphaFold3-predicted structures of individual filament proteins (bottom). These three branches converge to generate integrative atomic models of native *Leptospira* spp. filaments. Created in part with BioRender. Buschiazzo, A. (2026) https://BioRender.com/8s5vp7r

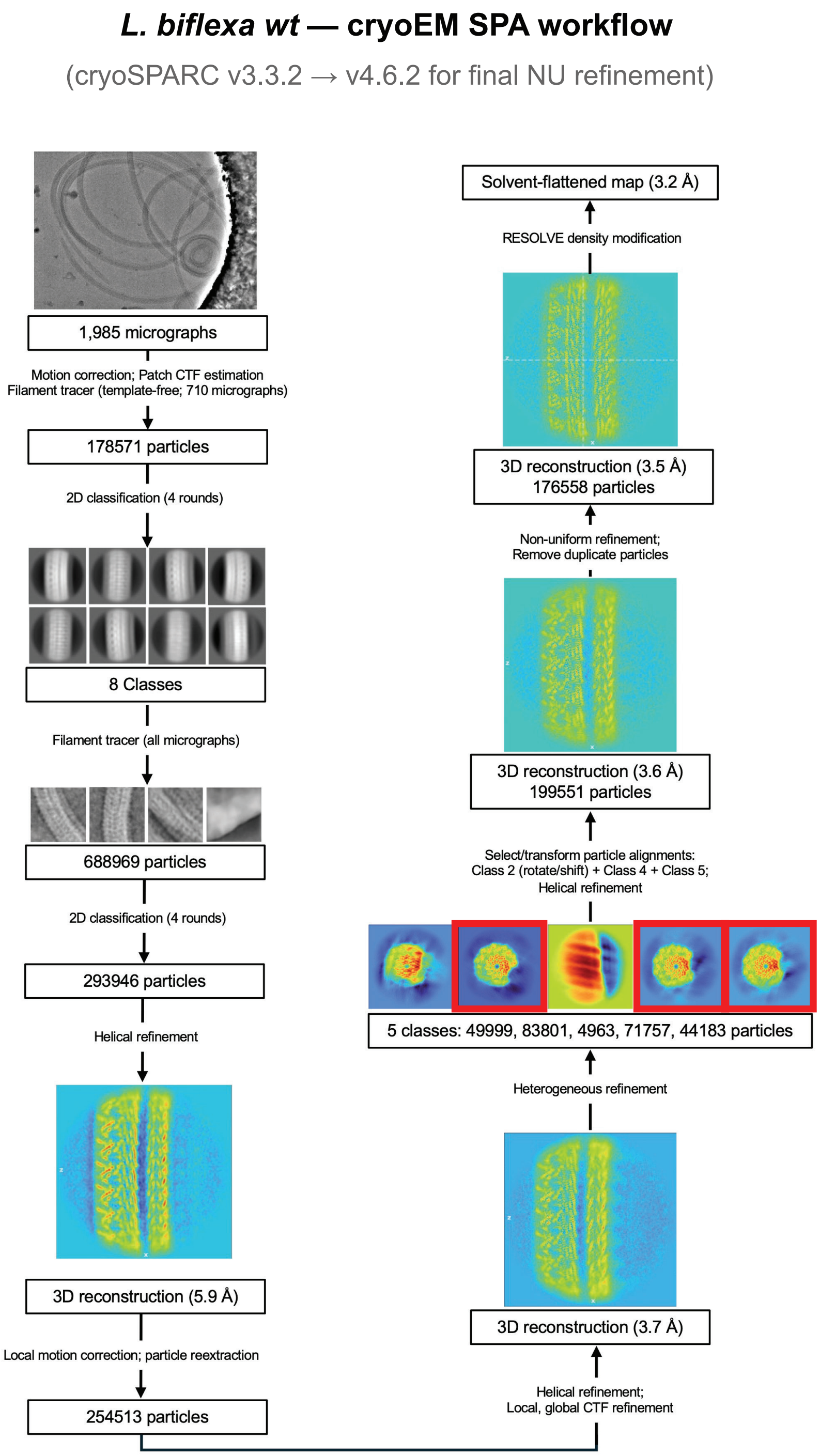

Supplementary Fig. 2. Cryo-EM single particle analysis workflow. 3D reconstruction workflow to obtain the structure of wild-type *Leptospira biflexa* filaments.

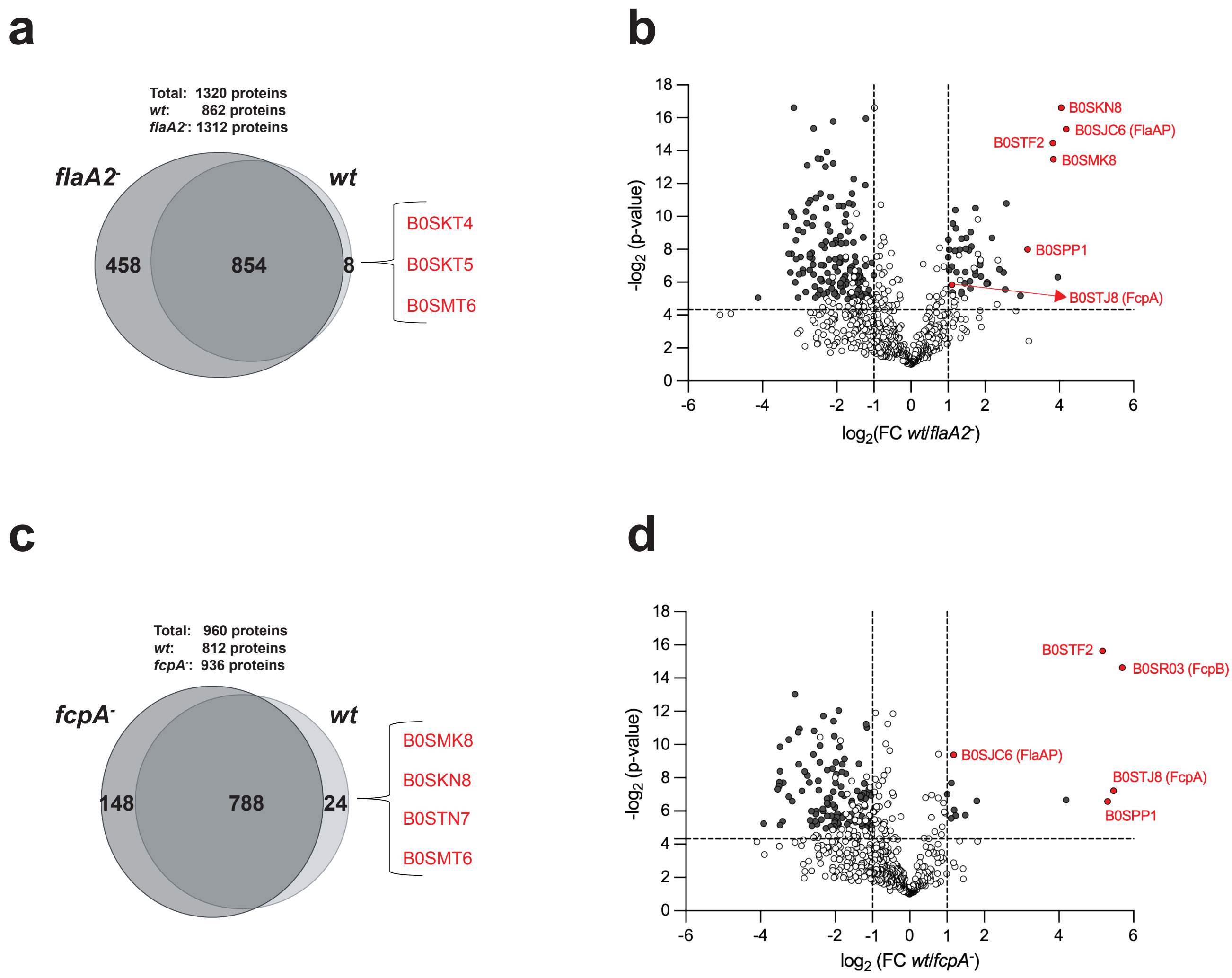

Supplementary Fig. 3. Label-free comparative proteomics of L. biflexa filaments from wt and sheath mutant strains. (a) Venn diagram comparing proteins identified in enriched filament preparations from *L. biflexa wt* and *flaA2⁻* strains (Bayesian probability, $p < 0.05$ [1]). Selected filament proteins identified in our structures and exclusively detected in wt are listed, with gene names in parentheses where annotated. **(b)** Volcano plot describing differential expression of proteins present in both *L. biflexa wt* and *flaA2⁻* strains. Each dot represents a protein, with darker dots being significantly differential ($|\log2FC| \geq 1$; $p \leq 0.05$). Proteins with altered expression also identified within the cryo-EM structure of *L. biflexa* filaments, are flagged in red and labelled with UniProt IDs (gene names for those annotated). See Supplementary Table 3 for detailed information. **(c)** Venn diagram comparing *L. biflexa wt vs fcpA⁻*, as in (a). **(d)** Volcano plot following same colour/labelling scheme as in (b).

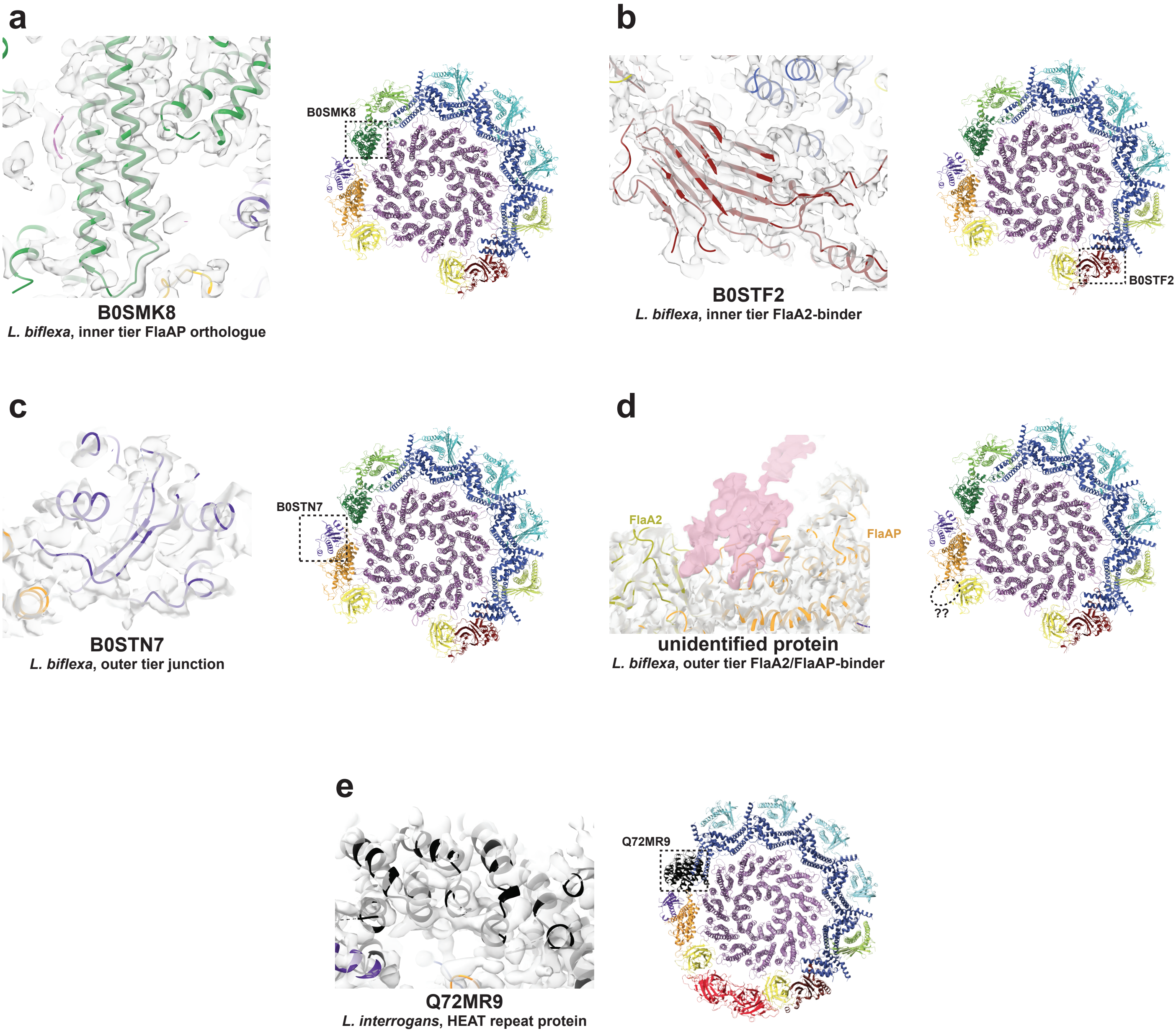

Supplementary Fig. 4. Validation of novel sheath protein assignments. Representative examples of newly identified sheath proteins fitted into cryo-EM density (contoured at 0.05 level); all consistent with interprotein crosslinks detected by mass spectrometry (see Table 1). Left: cryo-EM densities (semi-transparent surface) with refined models (cartoon). Right: position within the filament cross-sections (dashed boxes). **(a)** B0SMK8 (green) adopts a FlaAP-like fold in the *L. biflexa* inner tier. B0SMK8 interacts directly with the flagellin core, quasi-equivalent to FlaAP (B0SJC6), of which it is a structural paralogue. (b) B0STF2 (brick red) associates with one of the FlaA2 protofilaments on the concave surface in *L. biflexa*, contributing to the asymmetric inner tier organization. (c) B0STN7 (purple) occupies the outer tier at the junction between FlaAP and B0SMK8 in *L. biflexa*. (d) Unidentified protein, capping FlaA2 and FlaAP in the *L. biflexa* outer tier. The cryo-EM volume corresponding to this component is coloured in pink (left), no atomic model could be fitted within. (e) Q72MR9 (black), a HEAT repeat protein unique to the *L. interrogans* sheath, directly contacts the FlaB1 core in the compositional plasticity zone. This protein is absent in FlaB4-dominated *L. biflexa* wt filaments, where the mixed B0SMK8/B0SMT6 protofilament occupies the equivalent position.

L. interrogans wt — cryoEM SPA workflow

(cryoSPARC v3.3.2 → v4.6.2 for final NU refinement)

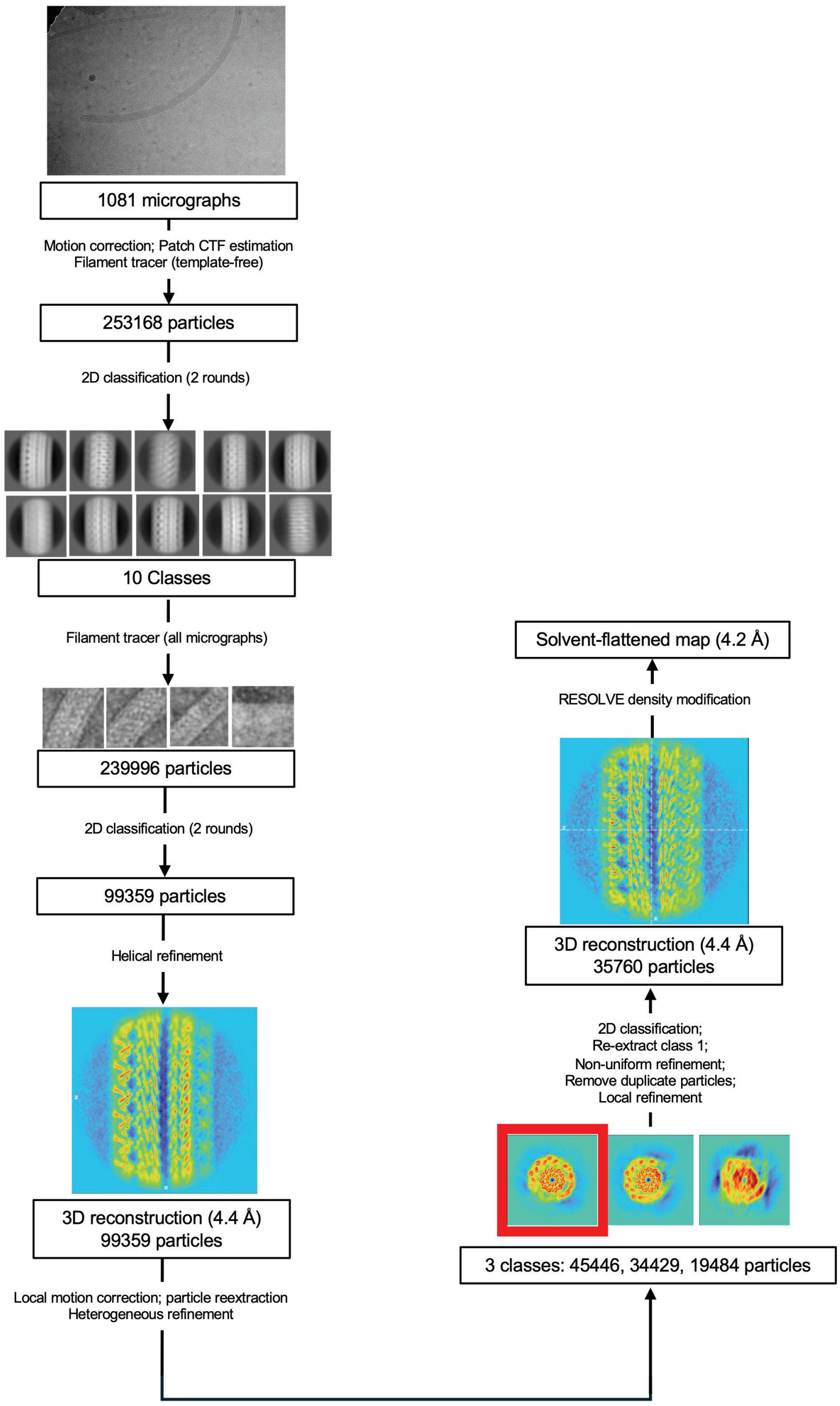

Supplementary Fig. 5. Cryo-EM single particle analysis workflow. 3D reconstruction workflow to obtain the structure of wild-type *Leptospira interrogans* filaments.

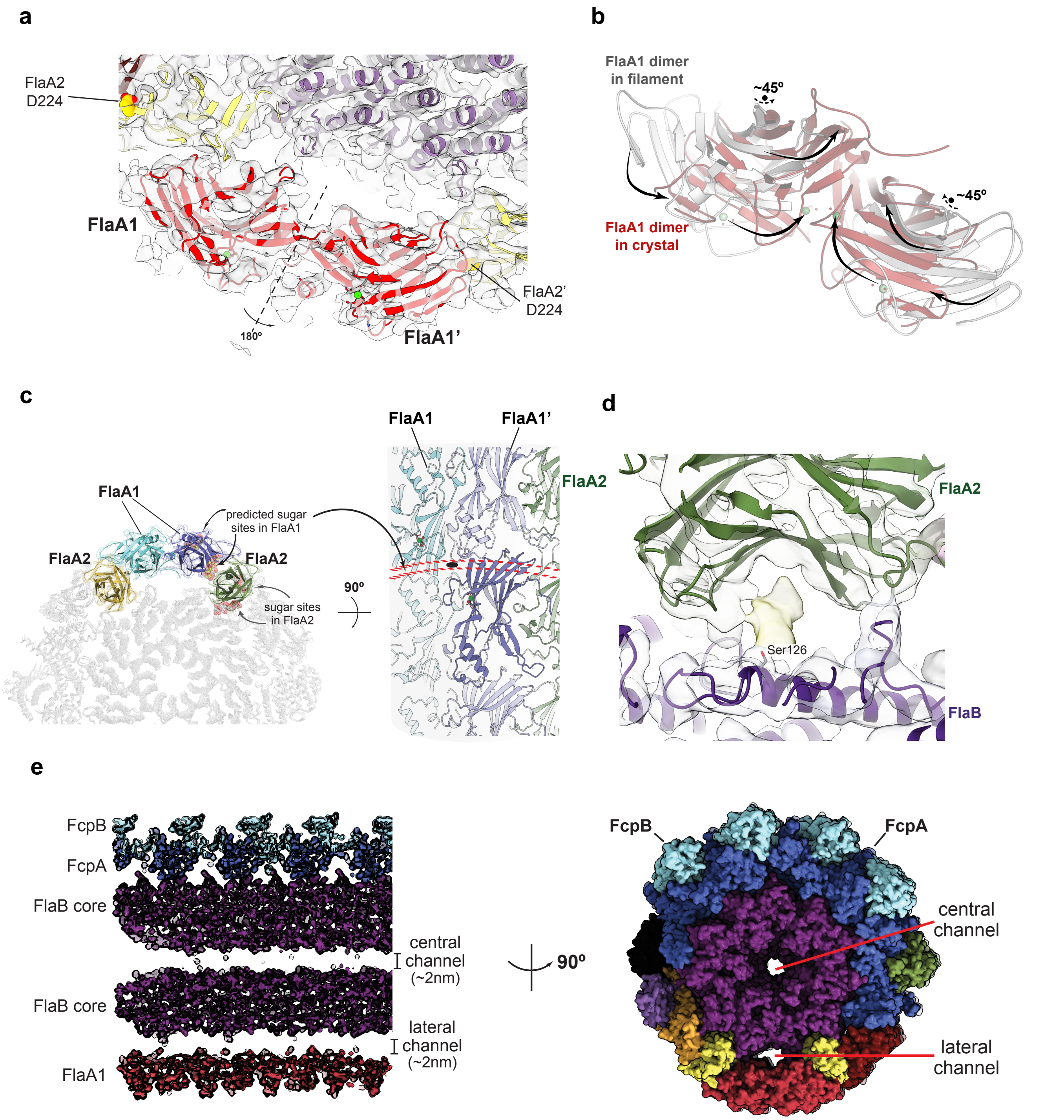

Supplementary Fig. 6. FlaA1 and FlaA2 in the assembled filament: differential orientation of carbohydrate-binding grooves. (a) *L. interrogans wt* cryo-EM map (semi-transparent) with fitted atomic model, viewed along the filament axis. FlaA1 (red) occupies the lateral groove as an antiparallel dimer related by a 2-fold axis perpendicular to the filament (marked). The non-equivalent FlaA1-FlaA2 interfaces on either side are indicated by a labelled residue on FlaA2. **(b)** Comparison of FlaA1 dimer geometry in the crystal vs in the assembled filament. In each case, dimer partners are related by a two-fold axis (yellow) that runs through a hydrophobic contact between a pair of symmetry-related leucines (Leu52; in the cryo-EM model it corresponds to Leu23 according to the mature protein numbering). The top monomers (red) have been aligned to match their orientations. To reach the filament geometry, dimer partners pivot away from each other by ~90° about the leucine contact, a hinge opening likely induced by interactions with flanking FlaA2 protofilaments. **(c)** Left: transverse view of *L. interrogans* cryo-EM structure showing the positions of the two FlaA2 protofilaments and the dimeric FlaA1, with putative sugar-binding sites indicated on both isoforms. Right: 90° rotated view revealing that the convex sugar-binding groove of each FlaA1 protomer faces outward; because the two protomers are related by a 2-fold axis (black ellipse), their individual grooves align to form a single continuous crevice spanning both subunits (red dotted lines; the two related FlaA1 protomers are highlighted in darker colours). **(d)** Close-up of *L. interrogans* FlaA2 engaging a FlaB1 *O*-glycan (Ser126) through its concave sugar-binding groove. The cryo-EM map (semi-transparent) shows clear glycan density. **(e)** The lateral channel beneath FlaA1 (~20 Å diameter), comparable in size to the central channel despite its flattened geometry.

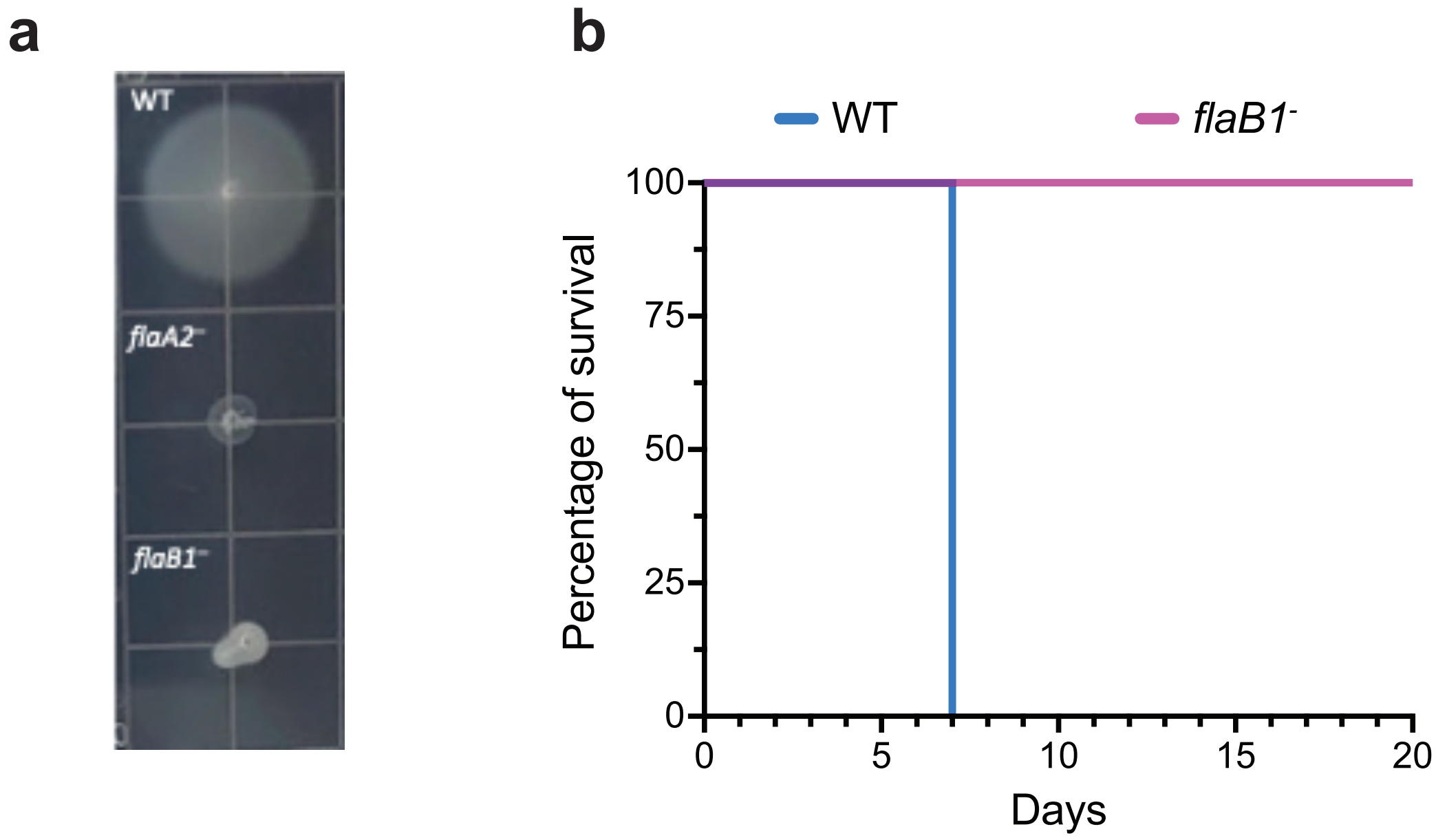

Supplementary Fig. 7. Extended phenotypic characterization of *L. interrogans flaB1*$^-$ mutant. **(a)** Representative images of dispersion halos in semi-solid agar for WT, *flaA2*$^-$ (non-motile control), and *flaB1*$^-$ strains after 10 days at 30°C. (b) Survival curves of Golden Syrian hamsters (n = 4 per group) infected intraperitoneally with 10^6 *L. interrogans* serovar Manilae WT or *flaB1*$^-$. All WT-infected animals succumbed by day 7, while *flaB1*$^-$-infected animals showed 100% survival throughout the 20-day observation period, demonstrating complete virulence attenuation.

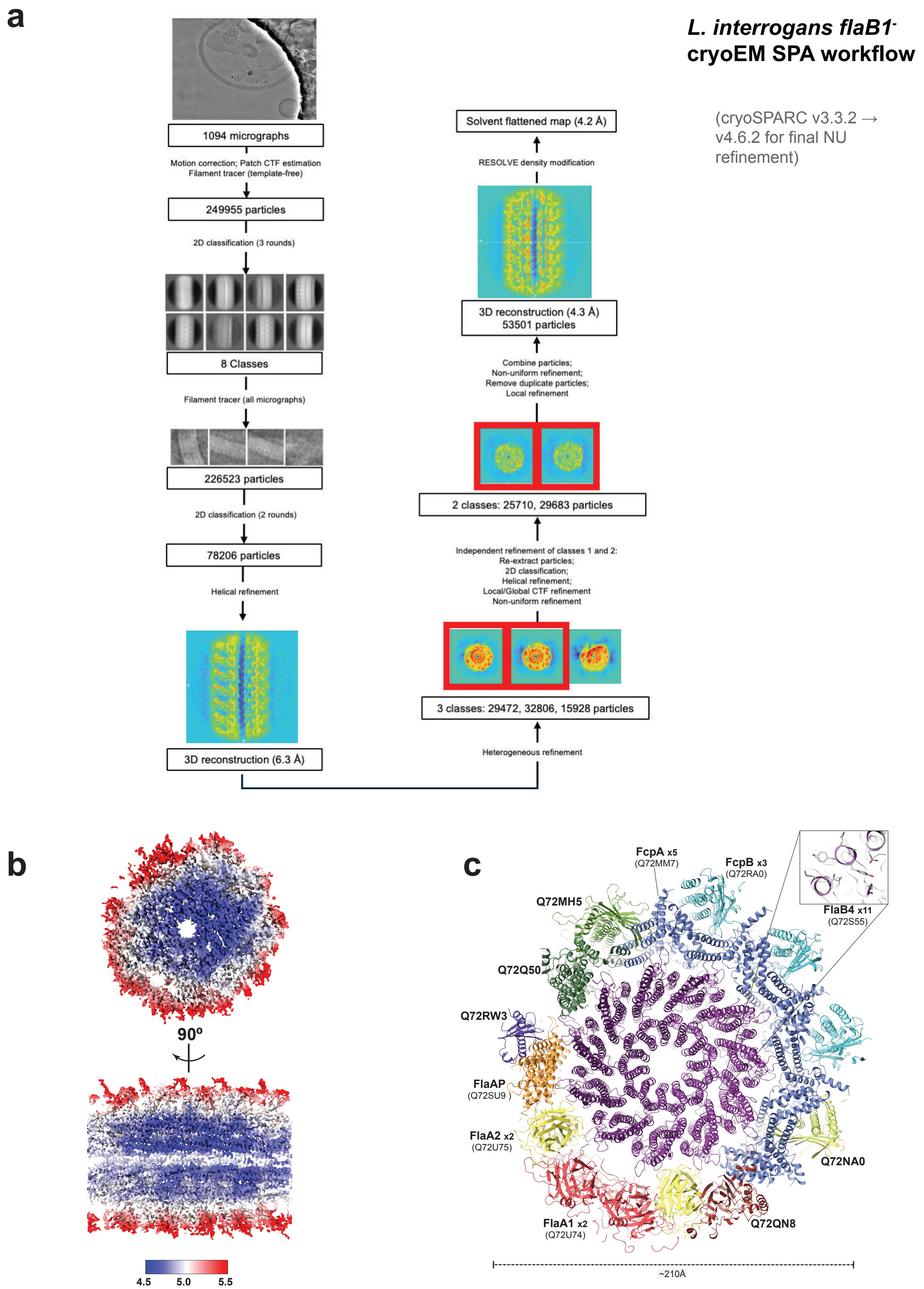

Supplementary Fig. 8. Cryo-EM structure of *Leptospira interrogans flaB1⁻* flagellar filament. (a) 3D reconstruction workflow to obtain the structure of *L. interrogans flaB1⁻* filaments by single particle analysis. **(b)** Cryo-EM map coloured by local resolution (scale bar: 4.5–5.5 Å), viewed from one end (top) and from the side (bottom), contoured at 0.1 threshold. **(c)** Refined atomic model shown as cartoon in transverse cross-section. Orthologous proteins are coloured and labelled as in Fig. 1. Inset: close-up of FlaB4 core density with fitted model.

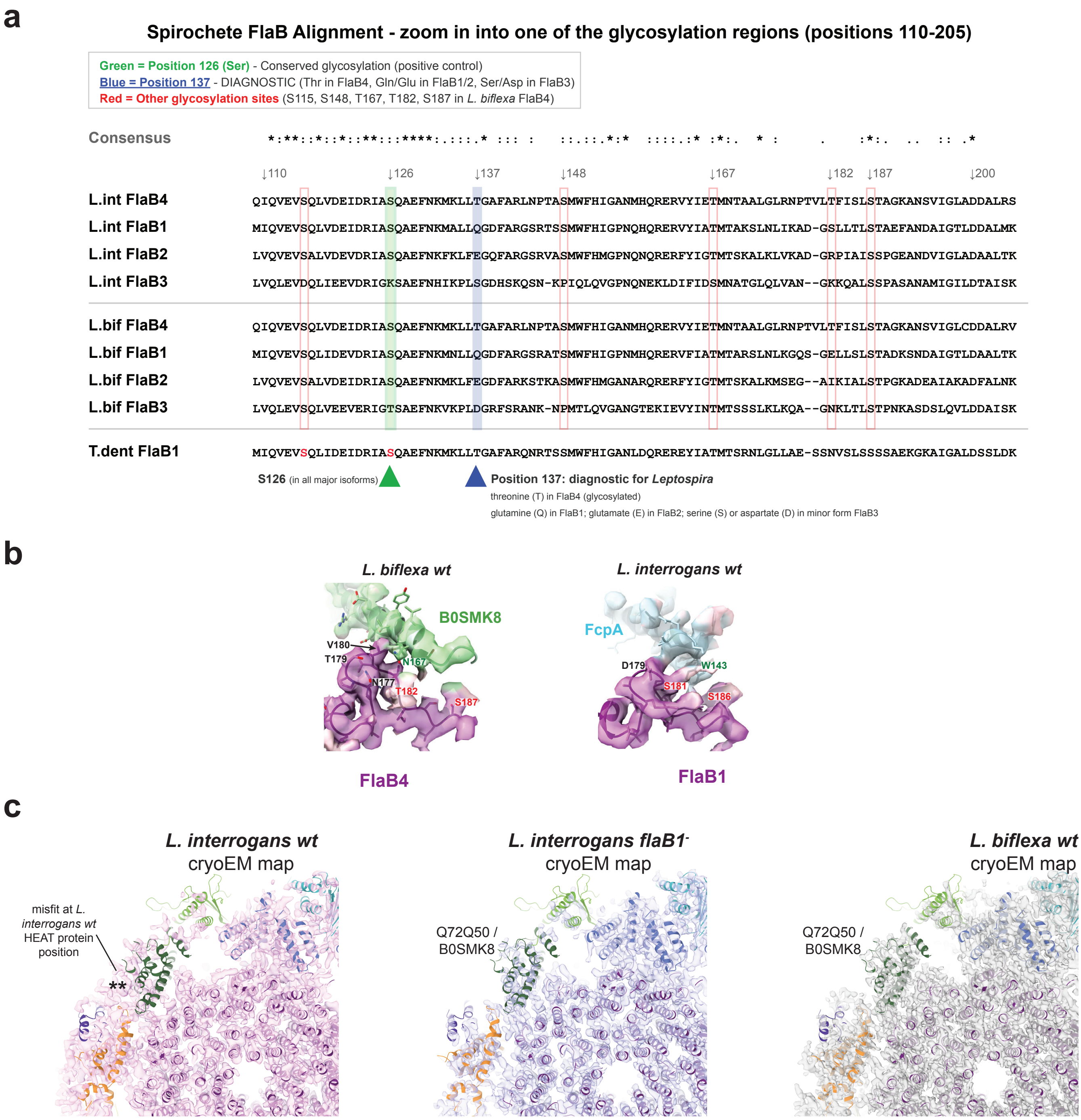

Supplementary Fig. 9. Core FlaB-induced variation of sheath architecture. (a) Multiple sequence alignment of *Leptospira* FlaB isoforms (L.int: *L. interrogans*; L.bif: *L. biflexa*) and *Treponema denticola* FlaB1 (T.dent), whose glycosylation sites have been determined experimentally [2]. The aligned fragment corresponds to a glycosylation-rich region (positions ~110–205). Diagnostic sites to distinguish FlaB isoforms are detailed. Note that for some sites, *O*-glycosylation is possible only in specific FlaB isoforms: among the most expressed isoforms, position 137 can only be glycosylated in FlaB4 (FlaB3 possesses a Ser that could accept an *O*-glycan, yet FlaB3 is a significantly minor component [3]). **(b)** FlaB isoform-dependent sheath contacts. In the *L. biflexa* structure (left), a hydrophobic contact is observed between FlaB4 V180 and N167 from sheath protein B0SMK8. This contact is also observed in the *L. interrogans flaB1⁻* structure (corresponding residues: FlaB4 V180 and sheath protein Q72Q50 N188), although features are less well resolved (not shown). Substitution of the FlaB4 V180-L181 residue pair by the single residue G180 in FlaB1 (see panel (a)) collapses the surrounding loop in our *L. interrogans wt* structure (right), indicating this contact is destabilized in the FlaB1-FlaB3 isoforms. Glycosylated residues and densities are labelled red/pink. **(c)** Comparison of cryo-EM densities at the compositionally variable sheath sector. The refined *flaB1⁻* atomic model (coloured as in Supplementary Fig. 8c) is shown fitted into three maps: *L. interrogans wt* (left), *L. interrogans flaB1⁻* (centre), and *L. biflexa wt* (right), all displayed at comparable contour levels. The model fits well into both *flaB1⁻* and *L. biflexa* maps. In contrast, the *L. interrogans wt* map shows additional density (asterisks) corresponding to the HEAT repeat protein Q72MR9 absent from the *flaB1⁻* and *L. biflexa* architectures, confirming that the mutant adopts a saprophyte-like sheath organization

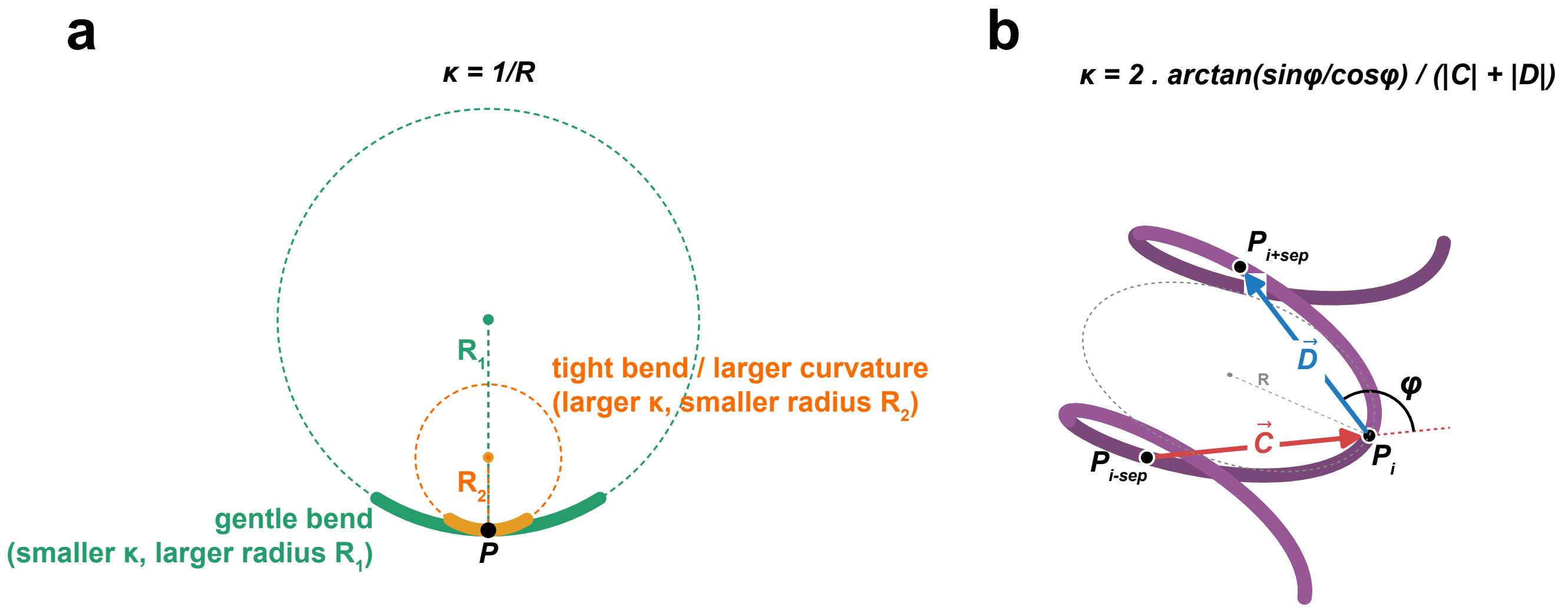

Supplementary Fig. 10. Definition of filament curvature κ. (a) In a planar curve, the curvature κ at a point P equals the inverse of the radius R of the osculating circle that best locally approximates the curve. A gentle bend corresponds to a large R and small κ (green); a tight bend to a small R and large κ (orange). The two curves shown share the same tangent point P and tangent direction, differing only in how tightly they bend (i.e. how curved they are). **(b)** For a 3D supercoiled trajectory such as the *Leptospira* filaments analysed in this work, the local osculating circle exists in a plane that re-orients along the path (faint dashed circle with radius R at point P_i; P_i corresponds to the position of one particle or asymmetric unit of the helical assembly); the Crenshaw algorithm [4] estimates κ directly from three consecutive trajectory points, spaced by a defined number of asymmetric units or separating (sep) particles. Vectors $C = P_i - P_{i-sep}$ and $D = P_{i+sep} - P_i$ are constructed; κ is then proportional to the angle φ between the extension of C and D, normalised by the sum of segment lengths: $\kappa = 2.\arctan(\sin\varphi/\cos\varphi) / (|C| + |D|)$. κ has units of inverse length; higher values indicate tighter local bending.

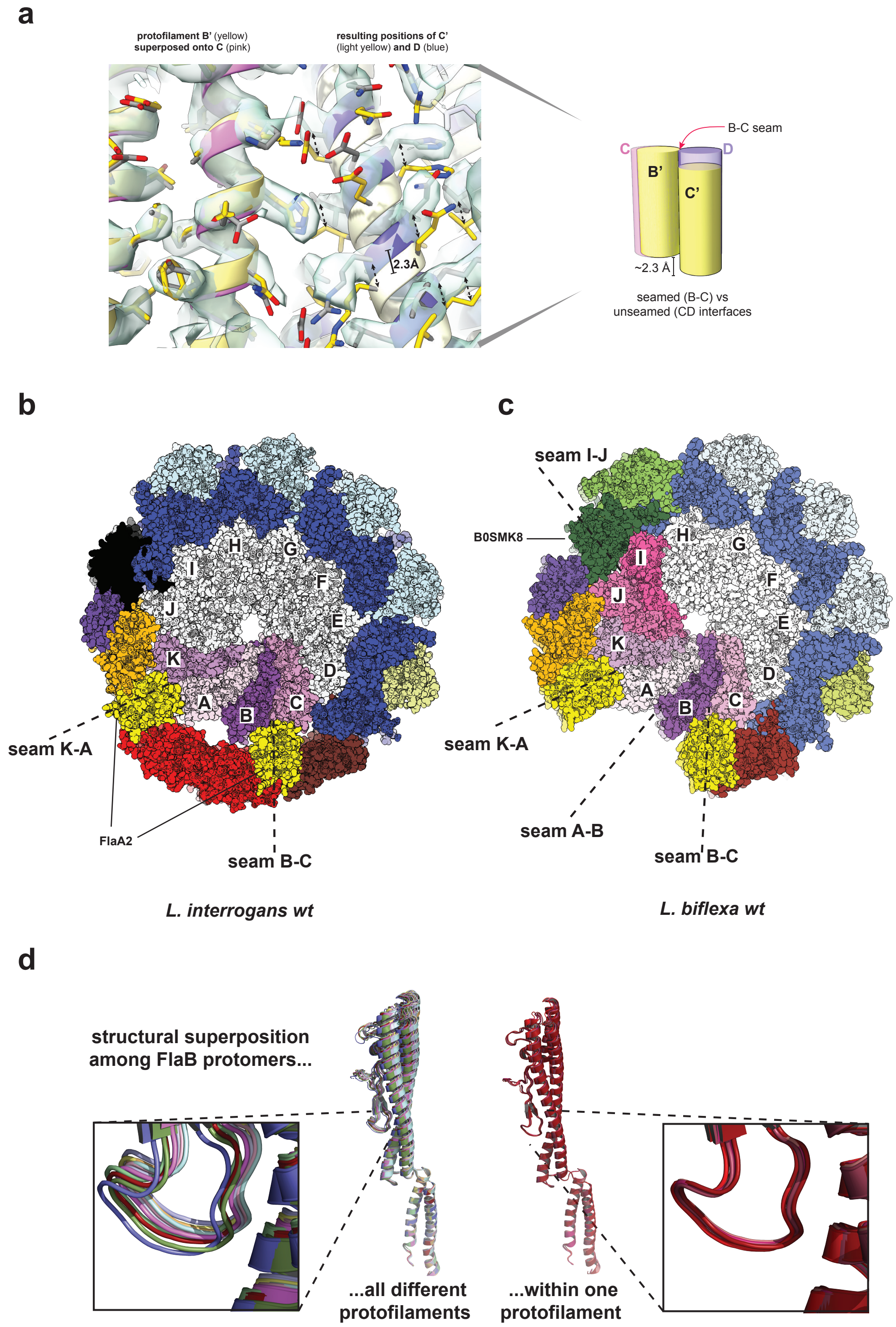

Supplementary Fig. 11. Seams and conformational flexibility in flagellin cores. (a) Seam detection by structural superposition. To reveal the translational discontinuity at a seamed interface, the seamed B′–C′ pair was superposed onto the seamless C–D pair by aligning chain B′ (yellow) onto chain C (pink) (left). If both interfaces were equivalent, their partners C′ (light yellow) and D (blue) would coincide; instead, C′ is displaced ~2.3 Å along the helical axis relative to D (right), revealing the seam. Cryo-EM density shown as semi-transparent surface. Schematic (far right) illustrates the protofilament sliding that defines seams. **(b)** *L. interrogans* filament cross-section with protofilaments labelled A-K. Seams at K-A and B-C (marked) lie beneath FlaA2 sheath moieties; seamless interfaces contact FcpA (blue). **(c)** *L. biflexa* filament cross-section indicating the location of four seams. Besides K-A and B-C, additional seams at A-B (beneath the FlaA1-absent position) and I-J (beneath B0SMK8) are marked. **(d)** Superposition of FlaB protomers reveals continuous conformational variation. Left: 11 protomers from successive protofilaments, all within one ~52 Å axial repeat; if the helical symmetry were perfect these would be identical, but they exhibit substantial conformational spread. Right: six protomers from a single protofilament (chains A3-A8) show much less variation. This continuous flexibility, rather than discrete L/R state switching, characterises *Leptospira* flagellin cores. The continuous conformational range among protofilaments is similar in *L. interrogans* (not shown).

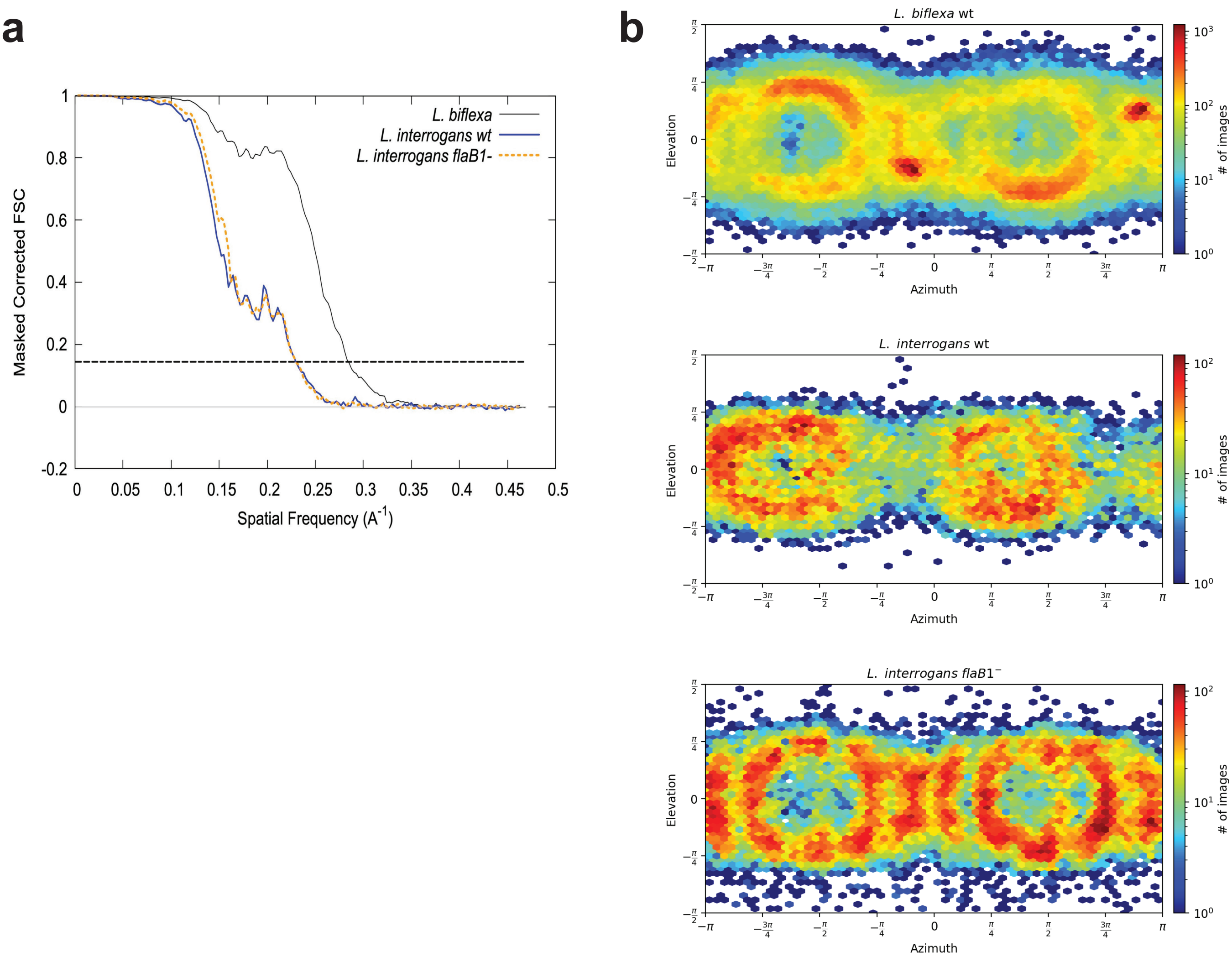

Supplementary Fig. 12. Overall cryo-EM data analyses for the three filament reconstructions. (a) Fourier Shell Correlation comparison of the three structures presented in the current work. Shown are data after application of the final CryoSparc-generated mask, corrected using high-resolution phase randomization. **(b)** Angular distribution of particle viewing directions for the final reconstructions of the three structures presented in the current work. Azimuth versus elevation (radians); hexagonal bins coloured by the number of particle images per orientation, on a logarithmic scale.

Supplementary Table 1. CryoEM data collection, processing and model refinement statistics.

	L. biflexa wt	*L. interrogans wt*	*L. interrogans flaB1⁻ mutant*
**Data collection & processing**			
Microscope	Titan Krios G2	Titan Krios G2	Titan Krios G2
Detector	Gatan K3	Gatan K3	Gatan K3
Magnification	81,000	81,000	81,000
Voltage (kV)	300	300	300
Electron exposure ($e^-/Å^2$)	25.7	61.5	61.5
Defocus range (µm)	-1.5 to -2.6	-1.5 to -2.6	-1.5 to -2.6
Pixel size (Å)	1.068	1.068	1.068
Symmetry imposed	C1	C1	C1
Number of Micrographs	1,985	1,081	1,094
Initial particle images (n)	688,969	239,996	226,523
Final particle images (n)	176,558	35,760	53,501
Resolution (Å) @ FSC threshold 0.143	3.5	4.4	4.3
after density modification	3.2	4.2	4.2
EMDB ID	EMD-75270	EMD-75268	EMD-75269
**Model refinement**			
Number of atoms			
All	248,736	248,288	253,725
Protein	248,706	248,234	253,671
Water	20	36	36
Ligand (Ca^{2+})	10	18	18
Model to map correlation			
CC(mask)	0.76	0.69	0.66
CC(volume)	0.72	0.65	0.62
CC(box)	0.62	0.54	0.51
RMSD			
Bond lengths (Å)	0.004	0.003	0.004
Bond angles (°)	1.07	0.81	1.09
Ramachandran statistics			
Favored (%)	97.59	95.07	94.91
Allowed (%)	2.34	4.51	4.75
Outliers (%)	0.07	0.41	0.33
Rama-Z score	0.95	-1.01	-0.58
Rotamer outliers (%)	3.36	0.34	0.03
C-beta outliers (%)	0	0.01	0.01
Clashscore	6.37	4.87	7.06
MolProbity overall score	1.84	1.60	1.74
PDB ID	pdb_000010LM	pdb_000010LK	pdb_000010LL

Supplementary Table 2. Protein components (UniProt ID codes) constituting the endoflagellar filament in *Leptospira*. Marked in bold red are the proteins actually identified and modelled within the cryoEM map for each *Leptospira* species (PDB IDs for the filament structures and the FlaA1 crystal structure are given in Supplementary Tables 1 & 3). Orthologues are included for completeness.

Protein	MW (kDa)	*L. biflexa* Uniprot ID	*L. biflexa* genome locus tag[1]	*L. interrogans* Uniprot ID	*L. interrogans* genome locus tag[2]	Copies/cell *L. interrogans*[3]	Filament localization
FlaB1	31	B0SSZ5 ¶	LEPBI_I2133	**Q72R58**	**LIC_11890**	24,000	Core flagellin
FlaB4	31	**B0SQZ5**	**LEPBI_I1589**	Q72S55 ¶	LIC_11531	7,160	Core flagellin
FlaB2	31	B0SSZ4¶	LEPBI_I2132	Q72R59¶	LIC_11889	2,800	Core flagellin
FlaB3	31	B0SS86¶	LEPBI_I1872	Q72S54¶	LIC_11532	200	Core flagellin
FcpA	36	**B0STJ8**	**LEPBI_I0267**	**Q72MM7**	**LIC_13166**	22,100	Sheath (convex)
FcpB	32	**B0SR03**	**LEPBI_I1597**	**Q72RA0**	**LIC_11848**	9,260	Sheath (convex)
FlaA1	36	B0SKT4**	LEPBI_I2335	**Q72U74**	**LIC_10788**	5,590	Sheath (concave)
FlaA2	27	**B0SKT5**	**LEPBI_I2336**	**Q72U75**	**LIC_10787**	6,020	Sheath (concave)
FlaAP	28	**B0SJC6**	**LEPBI_I0551**	**Q72SU9**	**LIC_11274**	870	FlaA2-associated
B0STF2	25	**B0STF2**	**LEPBI_I2297**	**Q72QN8**	**LIC_12075**	1,190	FlaA2-associated
B0SPP1	24	**B0SPP1**	**LEPBI_I3081**	**Q72NA0**	**LIC_12936**	300	Lateral region

Protein	MW (kDa)	*L. biflexa* Uniprot ID	*L. biflexa* genome locus tag[1]	*L. interrogans* Uniprot ID	*L. interrogans* genome locus tag[2]	Copies/cell *L. interrogans*[3]	Filament localization
B0STN7	22	**B0STN7**	**LEPBI_II0033**	Q75FY6 ¶ (replaced by paralogue **Q72RW3**)	LIC_20045 **(LIC_11625)**	150 (Q75FY6) 500 (Q72RW3)	Lateral region
B0SMK8	25	**B0SMK8 /** interspersed with paralogue **B0SMT6**	**LEPBI_I1029 / LEPBI_I2732**	Q72Q50 ¶ / Q72U89 ¶	LIC_12263 / LIC_10772	1,100	Lateral region
B0SKN8	25	**B0SKN8**	**LEPBI_I0662**	Q72MH5 ¶	LIC_13212	340	Lateral region
HEAT repeat	33	B0SNJ6 ¶	LEPBI_I1161	**Q72MR9**	**LIC_13123**	1,400	Lateral region

[1] *Leptospira biflexa* serovar Patoc strain Patoc 1 genome BioProject ID PRJNA20133 [5]
[2] *Leptospira interrogans* serovar Copenhageni strain Fiocruz L1-130 genome BioProject PRJNA10687 [6]
[3] Measured by Schmidt et al. [3]
¶ Not detected in the two wild-type filament structures solved in this study.
** Present in the MS data, yet systematically lost from the filament during sample preparation and absent from refined atomic model

Supplementary Table 3. X-ray data collection, processing and model refinement statistics.

	FlaA1
**Data collection**	
Beamline	Diamond Light Source I04
Wavelength (Å)	0.97951
Space group	$P2_12_12_1$
Cell dimensions	
a, *b*, *c* (Å)	81.4, 105.8, 108.7
α, β, γ (°)	90, 90, 90
Resolution (Å)	75.82–1.816 (1.848-1.816)*
Number unique reflections	85228 (4238)*
R_{merge}	0.106 (5.769)*
R_{pim}	0.030 (1.596)*
Mean I / σI	47.5 (0.5)*
$CC_{(1/2)}$	0.998 (0.375)*
Completeness (%)	100.0 (100.0)*
Redundancy	13.8 (14.0)*
**Refinement**	
Resolution (Å)	75.82–1.82 (1.84-1.82)*
Number reflections used in refinement	85179 (2640)*
Number reflections in the free set	4230 (146)*
R_{work} / R_{free}	0.184 / 0.215
Number of atoms (non-H)	5528
Protein	5073
Ions	38 (3 Ca^{2+} / 35 SO_4^{2-})
Water	417
Standard coordinate uncertainty (Å)	0.242
Average *B*-factor	50.15
R.m.s. deviations	
Bond lengths (Å)	0.017
Bond angles (°)	1.32
Ramachandran	
Favoured (%)	96.85
Allowed (%)	3.15
Outliers (%)	0.00
Rama-Z score	0.47
Clashscore	2.92
PDB ID	pdb_00009cyn

* Highest resolution shell

Supplementary Table 4. Orthologues/paralogues of *Leptospira* flagellar filament proteins. Proteins are grouped according to structural similarity identified by FoldSeek. Orthologous proteins (rows) are shown for 5 representative species. Paralogues, when identified, are listed within each Table cell. Proteins identified in *Leptospira* filaments are marked in bold. Low reliability hits are in italics (their structural similarities explain FoldSeek scores, but overall correspondence is not unambiguous as the other cases)

**Protein family**	**Orthologues** (in five representative species) **& paralogues** (within each cell)<br>**UniProt IDs**				
	Leptospira biflexa	*Leptospira interrogans*	*Treponema pallidum*	*Borrelia burgdorferi*	*Brachyspira hyodysenteriae*
FcpA-like	**B0STJ8**<br>B0SS28	**Q72MM7**<br>Q72NU5	*O83457***	ND‡	ND‡
FcpB-like	**B0SR03**<br>**B0SPP1**<br>**B0SKN8**<br>B0SSU6<br>-	**Q72RA0**<br>**Q72NA0**<br>Q72MH5<br>-<br>Q72UI5	ND‡	ND‡	ND‡
B0STN7-like	**B0STN7**<br>B0SR41	Q75FY6<br>**Q72RW3**	ND‡	ND‡	ND‡
B0SNJ6-like	B0SNJ6	**Q72MR9**	ND‡	ND‡	ND‡
FlaA-like	**B0SKT4**<br>(FlaA1)<br>**B0SKT5**<br>(FlaA2)<br>B0SPB9<br>B0SMM5	**Q72U74** (FlaA1)<br>**Q72U75** (FlaA2)<br>Q72Q36<br>Q72MZ0<br>Q72UQ4	P18193 (FlaA)<br>O83670<br>(FlaA2)	P70856	A0A3B6W463<br>A0A3B6VR02<br>A0A3B6VWM6
FlaAP-like<br>(associated to one FlaA2 protofilament in *Leptospira*)	**B0SJC6**<br>(FlaAP)<br>**B0SMK8**<br>**B0SMT6**<br>B0SQV9<br>B0SPD8<br>B0STI0<br>B0SMK9	**Q72SU9**<br>(FlaAP)<br>Q72Q50<br>Q72U89<br>Q72VN4<br>Q72WC0<br>Q72TL5	*P07643***	ND‡	A0A3B6VTA9
B0STF2-like<br>(associated to the other FlaA2, opposite to FlaAP)	**B0STF2**	**Q72QN8**	O83478<br>(residues 60-169)	*O51431***	*A0A3B6VQC5***

*** low reliability prediction*

‡ ND not detected

Supplementary References

1. Carvalho, P.C., et al., *Analyzing marginal cases in differential shotgun proteomics.* Bioinformatics, 2011. **27**(2): p. 275–6.
2. Kurniyati, K., et al., *A novel glycan modifies the flagellar filament proteins of the oral bacterium Treponema denticola.* Mol Microbiol, 2017. **103**(1): p. 67–85.
3. Schmidt, A., et al., *Absolute quantification of microbial proteomes at different states by directed mass spectrometry.* Mol Syst Biol, 2011. **7**: p. 510.
4. Crenshaw, H.C., C.N. Ciampaglio, and M. McHenry, *Analysis of the three-dimensional trajectories of organisms: estimates of velocity, curvature and torsion from positional information.* J Exp Biol, 2000. **203**(Pt 6): p. 961–82.
5. Picardeau, M., et al., *Genome sequence of the saprophyte Leptospira biflexa provides insights into the evolution of Leptospira and the pathogenesis of leptospirosis.* PLoS One, 2008. **3**(2): p. e1607.
6. Nascimento, A.L., et al., *Comparative genomics of two Leptospira interrogans serovars reveals novel insights into physiology and pathogenesis.* J Bacteriol, 2004. **186**(7): p. 2164–72.
