# Supplementary material for: Core–sheath coupling controls flagellar curvature and motility in Leptospira
Source: Nat Commun. 2026 Jul 25;17:9075. doi: 10.1038/s41467-026-75995-6 (PMC13518957; doi:10.1038/s41467-026-75995-6)
Supplement: Supplementary file 2 [file 41467_2026_75995_MOESM2_ESM.pdf]

Description of Additional Supplementary Files:

Supplementary Data 1: Crosslinking mass spectrometry analysis of purified filaments from *Leptospira biflexa*. (XLSX)

Supplementary Data 2: Label-free comparative proteomics of *Leptospira biflexa* filaments from wt vs *flaA2*$^{-}$ or *fcpA*$^{-}$ mutant strains. (XLSX)

Supplementary Data 3: FlaB isoform quantification in flagellar filaments from *Leptospira biflexa* and *L. interrogans* by label-free proteomics. (XLSX)

Supplementary Movie 1: *L. interrogans flaB1*$^{-}$ mutant displays wild-type swimming behaviour in liquid medium. (MP4)

Supplementary Software 1: Home-made bash script to obtain filament coordinates from RELION star files (processed cryo-EM micrograph data).

Supplementary Software 2: Home-made bash script to apply the Crenshaw method to calculate filament curvature (kappa) and torsion (tau) from micrograph coordinates.

Supplementary Software 3: Home-made bash script to download AlphaFold-predicted proteins from entire proteomes of selected spirochete representative species.
The three scripts are also archived, together with the three raw curvature data files, at Zenodo (https://doi.org/10.5281/zenodo.21104577).
