# Supplementary material for: Core–sheath coupling controls flagellar curvature and motility in Leptospira
Source: Nat Commun. 2026 Jul 25;17:9075. doi: 10.1038/s41467-026-75995-6 (PMC13518957; doi:10.1038/s41467-026-75995-6)
Supplement: Supplementary file 3 [file 41467_2026_75995_MOESM8_ESM.pdf]

nature portfolio

Corresponding author(s): Charles V Sindelar
Alejandro Buschiazzo

Last updated by author(s): Jul 2, 2026

# Reporting Summary

Nature Portfolio wishes to improve the reproducibility of the work that we publish. This form provides structure for consistency and transparency in reporting. For further information on Nature Portfolio policies, see our Editorial Policies and the Editorial Policy Checklist.

## Statistics

For all statistical analyses, confirm that the following items are present in the figure legend, table legend, main text, or Methods section.

| n/a | Confirmed | |
|---|---|---|
| [ ] | [x] | The exact sample size (*n*) for each experimental group/condition, given as a discrete number and unit of measurement |
| [ ] | [x] | A statement on whether measurements were taken from distinct samples or whether the same sample was measured repeatedly |
| [ ] | [x] | The statistical test(s) used AND whether they are one- or two-sided<br>*Only common tests should be described solely by name; describe more complex techniques in the Methods section.* |
| [x] | [ ] | A description of all covariates tested |
| [ ] | [x] | A description of any assumptions or corrections, such as tests of normality and adjustment for multiple comparisons |
| [ ] | [x] | A full description of the statistical parameters including central tendency (e.g. means) or other basic estimates (e.g. regression coefficient) AND variation (e.g. standard deviation) or associated estimates of uncertainty (e.g. confidence intervals) |
| [ ] | [x] | For null hypothesis testing, the test statistic (e.g. *F*, *t*, *r*) with confidence intervals, effect sizes, degrees of freedom and *P* value noted<br>*Give P values as exact values whenever suitable.* |
| [x] | [ ] | For Bayesian analysis, information on the choice of priors and Markov chain Monte Carlo settings |
| [x] | [ ] | For hierarchical and complex designs, identification of the appropriate level for tests and full reporting of outcomes |
| [ ] | [x] | Estimates of effect sizes (e.g. Cohen's *d*, Pearson's *r*), indicating how they were calculated |

*Our web collection on statistics for biologists contains articles on many of the points above.*

## Software and code

Policy information about availability of computer code

| | |
|---|---|
| Data collection | - SerialEM: open-source oftware for cryo-electron microscopy (cryo-EM) data acquisition.<br>- XDS: X-ray diffraction data collection<br>- AlphaFold3 server : 3D atomic structure prediction of target sequences |
| Data analysis | - Aimless : X-ray diffraction data processing (scaling and merging)<br>- CTruncate : X-ray diffraction data reduction from measured intensities to structure factor amplitudes<br>- Phaser : estimation of initial structure factor phases by molecular replacement<br>- Coot 1.1.18 : atomic model (re)building; real space refinement for both X-ray and cryoEM structures; and structural analyses<br>- ProSMART and phenix.secondary_structure_restraints : semi-automated pipelines to add extra restraints to atomic models (according to secondary structures, initial coordinates, etc) before refinement<br>- Phenix.refine : X-ray derived reciprocal space model refinement agasint measured X-ray structure factors<br>-phenix.real_space_refine : for real space refinemt of atomic models to maximize cross-correlation with cryoEM maps as data<br>- Servalcat: reciprocal space refinement of atomic models with cryoEM-data<br>- Checkmysequence v1.5.3: program to automate the pinpointing of potential errors in model building on cryoEM maps<br>- molprobity server: atomic model validation<br>- Phenix comprehensive validation cryoEM maps and models<br>- Phenix validation tools for crystallographic data and model<br>- CryoSPARC versions 3.3.2 and 4.6.2: for cryoEM data processing, refinement and 3D reconstruction (several programs implemented within CryoSPARC, such as motioncorr3, patch CTF estimation/correction, filament tracer partcile picking, etc)<br>- Phenix resolve program: to perform cryoEM map density modification procedures for post-processing |

- ChimeraX : model refinemnt with Isolde within, structural analyses, and model fitting into cryoEM maps
-FoldSeek remote server and locally run on our computing server : to find similar structures to the query, adjusting search parameters
- clustalW: to perform multiple sequence alignments within clustered families
- PatternLab for Proteomics V : analysis of mass spectrometry data
-MaxQuant: analysis of mass spectrometry data
- PyMOL/PyXlinkViewer: to map validated crosslinked residue pairs onto structural models
- xiVIEW : to visualize crosslinked interaction networks
- GraphPad Prism version 9.5.0: to analyze data from bacterial motility and epithelical translocation assays data and estimate statistical significance figures

For manuscripts utilizing custom algorithms or software that are central to the research but not yet described in published literature, software must be made available to editors and reviewers. We strongly encourage code deposition in a community repository (e.g. GitHub). See the Nature Portfolio guidelines for submitting code & software for further information.

## Data

Policy information about availability of data

All manuscripts must include a data availability statement. This statement should provide the following information, where applicable:

- Accession codes, unique identifiers, or web links for publicly available datasets
- A description of any restrictions on data availability
- For clinical datasets or third party data, please ensure that the statement adheres to our policy

Atomic coordinates of filament models and corresponding cryo-EM maps have been deposited in the worldwide Protein Data Bank under accession codes pdb_000010LM / EMD-75270 (L. biflexa wt); pdb_000010LK / EMD-75268 (L. interrogans wt); pdb_000010LL / EMD-75269 (L. interrogans flaB1-). Atomic coordinates and X-ray diffraction structure factors have been deposited in the wwPDB under accession code pdb_00009cyn (FlaA1 crystal structure). The mass spectrometry proteomics data have been deposited to the ProteomeXchange Consortium via the PRIDE [62] partner repository with the dataset identifiers PXD072058 (for L. biflexa wt and flaA2- strains); PXD030741 (L. biflexa fcpA- strain) [15]; PXD077813 (L. interrogans flaB1- strain); and PXD073467 (for crosslinking data). Whole genome sequencing data of generated Leptospira mutant strains are available at NCBI under BioProject accession # PRJNA1416034. The filament curvature raw data for the three strains studied in this work are available at Zenodo under https://doi.org/10.5281/zenodo.21104577.

## Research involving human participants, their data, or biological material

Policy information about studies with human participants or human data. See also policy information about sex, gender (identity/presentation), and sexual orientation and race, ethnicity and racism.

| | |
|---|---|
| Reporting on sex and gender | N/A |
| Reporting on race, ethnicity, or other socially relevant groupings | N/A |
| Population characteristics | N/A |
| Recruitment | N/A |
| Ethics oversight | N/A |

Note that full information on the approval of the study protocol must also be provided in the manuscript.

# Field-specific reporting

Please select the one below that is the best fit for your research. If you are not sure, read the appropriate sections before making your selection.

[x] Life sciences [ ] Behavioural & social sciences [ ] Ecological, evolutionary & environmental sciences

For a reference copy of the document with all sections, see nature.com/documents/nr-reporting-summary-flat.pdf

# Life sciences study design

All studies must disclose on these points even when the disclosure is negative.

| | |
|---|---|
| Sample size | Number of X-ray diffraction indexed reflections were chosen to maximize data set completeness according to standard data collection best practices. For cryo-EM, particle numbers reflect the valid particles retained after classification; these were maximized to obtain the highest gold-standard FSC resolution achievable given the filaments' conformational flexibility. Number of spectra in the MS analyses were sufficient to yield statistically significant comparative figures; for the cross-linking interactomics, sample amounts were set so that the yield of purified cross-linked material was suitable for downstream MS analyses. Swimming velocity was measured on ~60 cells per condition; semi-solid agar dispersion used n = 6 independent experiments; epithelial translocation used three independent experiments each with three technical replicates; hamster infection used n = 4 per group. Curvature was quantified from 20,977–55,579 per-filament measurements across 309–1,148 filaments per strain. These numbers were sufficient to yield consistent effects with clear separation between groups. |
| Data exclusions | No data were excluded |
| Replication | The main approaches throughout this work (cryoEM, protein crystallography and MS) are methods that imply large number of replicas such |

| | |
|---|---|
| | that the final numbers that are being analyzed result from the averaging of large sets of individual measurements. They have thus high redundancy/multiplicity data structures on their own.<br>For the live Leptospira cells assays: motility results were mean figures from 60 bacteria (for swimming velocity estimations) , or triplicate experiments for dispersion in solid media; epithelial translocation assays were performed by triplicate in each of three independent experiments; animal infections were performed with 4 independent animals per group. |
| Randomization | Experimental groups were defined by bacterial genotype (wild-type versus specific mutants) rather than by allocation of subjects, so randomization of samples into groups does not apply; all strains were cultured and assayed in parallel under identical conditions. For hamster infections, animals of matched age and source were assigned to the wild-type or mutant inoculum groups; no randomization procedure was used, and sex, husbandry, age and handling were kept identical across groups to control covariates. |
| Blinding | Investigators were not blinded to group allocation. For the structural, proteomic and curvature analyses, blinding is not applicable, as data collection and processing are instrument- and algorithm-based and group identity does not enter the automated pipelines. For the motility, epithelial-translocation and hamster-survival experiments (Fig. 4, Supplementary Fig. 7), strains were not blinded; the primary readouts are quantitative and objectively scored (automated particle tracking, chamber counts, survival), which limits the scope for observer bias. |

# Reporting for specific materials, systems and methods

We require information from authors about some types of materials, experimental systems and methods used in many studies. Here, indicate whether each material, system or method listed is relevant to your study. If you are not sure if a list item applies to your research, read the appropriate section before selecting a response.

## Materials & experimental systems

| n/a | Involved in the study | |
|---|---|---|
| ☒ | ☐ | Antibodies |
| ☒ | ☐ | Eukaryotic cell lines |
| ☒ | ☐ | Palaeontology and archaeology |
| ☐ | ☒ | Animals and other organisms |
| ☒ | ☐ | Clinical data |
| ☒ | ☐ | Dual use research of concern |
| ☒ | ☐ | Plants |

## Methods

| n/a | Involved in the study | |
|---|---|---|
| ☒ | ☐ | ChIP-seq |
| ☒ | ☐ | Flow cytometry |
| ☒ | ☐ | MRI-based neuroimaging |

# Animals and other research organisms

Policy information about studies involving animals; ARRIVE guidelines recommended for reporting animal research, and Sex and Gender in Research

| | |
|---|---|
| Laboratory animals | Golden Syrian hamsters (RjHan:AURA, Janvier Labs) |
| Wild animals | N/A |
| Reporting on sex | Only male individuals were used, to reduce variability in Leptospira virulence effects. |
| Field-collected samples | N/A |
| Ethics oversight | All animal procedures were approved by the Institut Pasteur Animal Care and Use Committee (CETEA #220016) and the French Ministry of Agriculture, in accordance with European Union Directive 2010/63/EU for the protection of animals used for scientific purposes. |

Note that full information on the approval of the study protocol must also be provided in the manuscript.

# Plants

| | |
|---|---|
| Seed stocks | N/A |
| Novel plant genotypes | N/A |
| Authentication | N/A |
