# Supplementary material for: Core–sheath coupling controls flagellar curvature and motility in Leptospira
Source: Nat Commun. 2026 Jul 25;17:9075. doi: 10.1038/s41467-026-75995-6 (PMC13518957; doi:10.1038/s41467-026-75995-6)
Supplement: Supplementary file 4 [file 41467_2026_75995_MOESM9_ESM.pdf]

nature portfolio

# Peer Review File

# Core–sheath coupling controls flagellar curvature and motility in *Leptospira*

Corresponding Author: Dr Alejandro Buschiazzo

**This file contains all reviewer reports in order by version, followed by all author rebuttals in order by version.**

Version 0:

Reviewer comments:

Reviewer #1

(Remarks to the Author)
Spirochetes possess a highly intricate mode of motility that enables cells to move efficiently through viscous or structured environments and is crucial for bacterial pathogenicity. These cells harbour bipolar flagella that are located within the periplasm rather than extending into the extracellular space. Flagellar activity induces morphological changes in the cell envelope that are translated into propagating helical waves, thereby driving forward movement. Earlier studies have demonstrated that spirochetal flagellar filaments have a complex architecture, consisting of a glycosylated inner filament surrounded by a proteinaceous sheath composed of multiple proteins. More recent work has provided increasing insight into the structures of individual components and of the filament as a whole; however, a comprehensive model that integrates all components is still lacking. In the present study, the authors performed a thorough analysis of the flagella of several species of the genus Leptospira, including selected mutants, combining in silico and in vitro structural approaches with cryo-electron tomography and physiological analyses. Taken together, this work represents a major step forward in our understanding of the structure and function of this remarkable molecular machine.

In my opinion, the structural analyses are highly impressive and largely comprehensive. I nevertheless have several suggestions regarding clarification and presentation.

Lines 119 and 123 (and throughout the manuscript): The authors frequently refer to glycosylation of the FlaB subunits and to the importance of this modification for interactions with the sheath. It would be helpful if this feature could be illustrated at least once in a way that is readily accessible to non-specialist readers (for example in Figure 1).

Line 143: What are the major differences between the respective proteins? Are particular domains missing at the N- or C-terminus? Please clarify this point more explicitly.

Line 164: It might already be appropriate here to note that FlaA1 is expected to localise to the gap within the sheath.

Line 232: What would be the expected structural and physiological consequences of a substitution to serine at this position? Addressing this experimentally could provide a particularly informative addition.

A final remark: Two recent studies now provided high resolution structures of the Treponema flagella (Wang et al., doi: 10.64898/2026.01.31.703064) and Leptospira (Kawamoto et al, DOI: 10.1038/s44318-026-00731-1). I would ask the authors to amend their discussion to include at least the major findings of these studies.

Reviewer #2

(Remarks to the Author)
This paper describes the structure and function of spirochete flagella, including mechanistic detail of how curvature relates to swimming ability in varied viscosities.

The work is of signifiance to the fields of spirochetes, pathogenesis, and methodology for image analysis of cryo-EM data

The work supports the authors' conclusions, with sound methodology that meets current standards, with enough detail for reproduction.

Specific comments:

L64. remove the word ''while' and start a new sentence.
L70 'unique adaptations' -- from what are they adapted?
L108-109 'one additional unidentified component, distributed with a
radially asymmetric organization and a ~52 . periodicity along the filament (Fig. 1b,d).'
is it the sheath or the unidentified component that is radially asymmetric and 52A periodicity? these figures do not show this.
L120 what is a 'heterogeneous organization'? you have a high resolution structure, so the organization must not be heterogeneous
L130-131 'density for one additional outer-tier component, capping FlaA2 and FlaAP, could
131 not be interpreted' where is this in the figures? there should be density maps... -- the only density shown is of 3 amino acids in fig 1d.
L145-148. It is difficult to relate the text to the figure, which discusses the structure in terms of chains A,B,C. Where is the jelly roll like beta sandwich?
Extended Figure 6a shows the concave and convex faces, which are not visible in Figure 2
Extended Figure 6b is not interpretable

L163-164 'Whether FlaA1 is incorporated within native
filaments remained to be determined.' this is confusing -- is L. borgpetersenii a native filament?
L170-171 please clarify 'further density modification'. Extended Data Fig 5 shows a final resolution of 4.4, not 4.2 as stated in the text.
Extended Data Fig 6b -- the drawing appears to indicate that each subunit rotates 45 degrees, for a total change of 90 degrees, however the text indicates the total change is 45 degrees; which is correct?

L245-253 -- is there a way to describe what 'quantitative curvature' measures that results in units of inverse microns?

Table 1 - clarify 'Exclusive' and define 'FC'
L190 only Fig 3b (not 3c) shows 9 distinct proteins.

L834 - "11 protomers from different protofilaments at one axial level" this is a helical filament, so they are not at 'one axial level'

L266-268 "revealing an integrated architectural
system that tunes flagellar mechanical properties essential for Leptospira motility in
environments of varying viscosity." is discussion, not results.

L245. an explanation of what a curvature of x/micron means would be helpful.

L305-306. using delta k here is confusing, as it was used as an error measurement above.

L339-340 "This suggests that additional/alternative components
340 may be incorporated at different lifecycle stages or at precise filament locations."
This is a stretch of what one can say from the data. To validate this, one would have to have synchronous cultures and do LC-MS at specific cell-cycle timepoints.

L552 - were the map data collection and resulting resolution differences due solely to the dataset sizes ( if this is not known, it does not need to be addressed).

Reviewer #3

(Remarks to the Author)
In this manuscript by San Martin et al., the atomic structure of the Leptospira flagellar sheath and its relationship with the core has been resolved by a number of approaches, including cryoEM, xray crystallography, crosslinking mass spectrometry, and mutational analysis. The study is comprehensive and provide new insights into the multiple proteins involved and the complex asymmetric nature of the structure. Some issues need to be addressed, as indicated in the comments below.

1. l. 117-119 and elsewhere. It is stated that FlaB4 is the major flagellar core component in L. biflexa, and FlaB1 is the dominant FlaB paralogue in the L. interrogans serovar Manilae strain L495 examined. It is unclear how the ‘dominant’ core protein(s) could be determined by cryoEM alone, in that all the structures of the closely related core proteins would be averaged unless they are in a regular arrangement. The crosslinking MS studies show that FlaB4, FlaB1, and FlaB2 were crosslinked indicating that they were in close proximity, which seems contrary to the predominant presence of a single FlaB protein. Also, it seems unlikely that FlaB3 would not be incorporated into the flagella at all. The authors have ample MS data, which should be of value in determining the relative concentrations of the FlaB paralogues in the purified flagella from the wt organisms and flaB1 L. interrogans mutant. This information should be incorporated into the manuscript and the interpretation of the core composition reevaluated accordingly.

2. Please provide a supplemental table indicating the genome sequences used and the gene designations and PDB numbers for each of the proteins described in this manuscript. In particular, the FlaB1 through FlaB4 designations are confusing and do not appear to match prior designations. Note that a newly annotated version of the L. biflexa Patoc 1 genome was released recently (NC_010602.1).
2. It should be clearly stated in the figure legends that the designations (e.g. BOSMK8) are for PDB accession numbers, if that is the case. (They do not as yet appear in the PDB.)

3. Leptospira are different from other spirochetes, in that the flagella are quite short. It would be worthwhile to include some discussion about this aspect and how the complex sheath may be important in providing in stiffening the flagella and (perhaps) promoting locomotion.

5. During swimming motion, the flagella at either end are rotating in opposite directions. Are there any theories as to how the thickness of the sheath may affect rotation in opposite directions? Did classification indicate different classes consistent with alterations in the structure?

6. Fig. 1 and associated text. The absence of FlaA1 from the L. biflexa filament structure may simply reflect that this association is less stable than in L. interrogans, resulting in its loss during purification.

7. l. 58 (minor). estimated to cause?

Reviewer #4

(Remarks to the Author)
The authors present three reconstructions of Leptospira flagellae, from two different species and a mutation that illuminates the differences in curvature between the wild types of each species.
The structures are well determined and modeled, with appropriate FSC behavior and model-to-map statistics for their resolution, as revealed in their PDB Validation Reports. The models are adequate to assign residue-level features, and clearly show the differences among the maps and models of the three structures.
Given the importance of “curvature” in this paper, it was difficult as a non-specialist to determine the exact physical meaning of curvature as they use it. The axis of the relevant plots is labeled in units of μm-1, so I assume it is something per micron. Is it superhelical waves per micron? A cartoon would help. The movies are not quite clear enough to see the superhelical corkscrew shape.

Version 1:

Reviewer comments:

Reviewer #1

(Remarks to the Author)
The authors have addressed all issues I had with their manuscript. Great Story.

Reviewer #2

(Remarks to the Author)
The authors have acceptably responded to the reviewers’ questions.

Reviewer #3

(Remarks to the Author)
The authors have very effectively addressed the concerns raised in the prior review. In particular, both citations and new mass spectroscopy data (Table S4) now substantiate the relative abundance of the four FlaB components in L. biflexa, L. interrogans, and mutant cell preparations. A paragraph has also been added to the Discussion that describes possible effects of the complex core-sheath structure on both rotational reversal and stability of the single, short flagellum found at each end of the cells. Additional information is also provided regarding the structural effects of FlaB glycosylation.

Reviewer #4

(Remarks to the Author)
The authors have clarified the points I raised.

**Open Access** This Peer Review File is licensed under a Creative Commons Attribution 4.0 International License, which permits use, sharing, adaptation, distribution and reproduction in any medium or format, as long as you give appropriate credit to the original author(s) and the source, provide a link to the Creative Commons license, and indicate if changes were made.
In cases where reviewers are anonymous, credit should be given to 'Anonymous Referee' and the source.
The images or other third party material in this Peer Review File are included in the article's Creative Commons license, unless indicated otherwise in a credit line to the material. If material is not included in the article's Creative Commons license and your intended use is not permitted by statutory regulation or exceeds the permitted use, you will need to obtain permission directly from the copyright holder.
To view a copy of this license, visit https://creativecommons.org/licenses/by/4.0/

## Reviewer 1

**R1.1** — *"Lines 119 and 123 (and throughout the manuscript): The authors frequently refer to glycosylation of the FlaB subunits and to the importance of this modification for interactions with the sheath. It would be helpful if this feature could be illustrated at least once in a way that is readily accessible to non-specialist readers (for example in Figure 1)."*

We thank the reviewer for this helpful suggestion. A new panel has been added to Figure 1 showing a schematic of the FlaB4 sequence, indicating the position of the *O*-glycan-modified Ser/Thr residues. Insets in this panel illustrate the 3D location of these sites in one FlaB4 protomer, and a representative region of the cryo-EM map showing the carbohydrate moieties.

**R1.2** — *"Line 143: What are the major differences between the respective proteins? Are particular domains missing at the N- or C-terminus? Please clarify this point more explicitly."*

Addressed. The paralogues share the same jelly-roll fold; the size difference between them maps onto loops within this fold, not onto terminal extensions or insertions of discrete domains.

**R1.3** — *"Line 164: It might already be appropriate here to note that FlaA1 is expected to localise to the gap within the sheath."*

Agreed. The sentence has been rewritten, also considering reviewers #3's comment: "Whether FlaA1 is incorporated as a structural component within assembled filaments, possibly occupying the unfilled groove between the two FlaA2 protofilaments, remained to be determined by direct structural analysis."

**R1.4** — *"Line 232: What would be the expected structural and physiological consequences of a substitution to serine at this position? Addressing this experimentally could provide a particularly informative addition."*

This is a thoughtful suggestion. The subtle point here is that a Thr→Ser substitution would preserve the glycosylation competence of the site, because *O*-glycosylation proceeds on the γ-hydroxyl group shared by both residues. The predicted effect would therefore be a minor local change (loss of the Cβ-methyl, ~10 Å² van der Waals contact; slightly altered rotameric preferences), rather than loss of glycan attachment. For the specific hypothesis being tested, more informative substitutions would be Thr→Ala, or Thr→Gln (mimicking the native residue of FlaB1 at the homologous position), both abolishing glycosylation, testing whether the glycan drives/affects sheath recruitment. Both mutations are planned as part of a dedicated biochemical follow-up. At this time however, and considering that systematic site-directed mutagenesis of individual FlaB paralogues in pathogenic *Leptospira* remains technically demanding, such a study falls beyond the scope of the present manuscript.

What we have further pursued, inspired by this reviewer's suggestion, is pinpointing other potential core-driven triggers of sheath rearrangement. We discovered isoform-specific differences in a nearby FlaB loop that directly contacts sheath protein B0SMK8/Q72Q50 (*L. biflexa*/*L. interrogans*, respectively), likely contributing to the exchange of this protein for FcpA when the core is switched to FlaB1. We revised this results paragraph (line 239 of the

original manuscript / line 273 in revised version) in this regard, and an Extended Data Figure panel (new Extended Data Fig. 9b) reporting this finding. We also made additional minor edits to improve the narrative flow of this section.

**R1.5** — *"A final remark: Two recent studies now provided high resolution structures of the Treponema flagella (Wang et al., doi: 10.64898/2026.01.31.703064) and Leptospira (Kawamoto et al, DOI: 10.1038/s44318-026-00731-1). I would ask the authors to amend their discussion to include at least the major findings of these studies."*

Agreed, these reports came out after we submitted our manuscript (actually, a second work on *T. denticola* besides Wang el al, was also communicated as a preprint). A dedicated paragraph has now been incorporated in the revised Discussion.

---

## Reviewer 2

**R2.1** — *"L64. remove the word 'while' and start a new sentence."*

Corrected.

**R2.2** — *"L70 'unique adaptations' — from what are they adapted?"*

The phrasing was insufficiently anchored. The implicit reference point is the canonical exoflagellar model, which the next sentence in the same paragraph contrasts against ("Unlike exoflagellated bacteria…"). To make the comparison explicit, the sentence at line 70 has been expanded to name one concrete adaptation per subsystem and the functional rationale, and now reads:

"…spirochetal motility is linked to distinctive features of their flagellar motor (including a periplasmic collar and additional stators), hook (with extensive inter-subunit cross-linking of FlgE), and filament (the proteinaceous sheath of additional proteins producing curved, stiff assemblies), collectively thought to support the higher torque loads typical of spirochete swimming [11, 12]. Of these features, the filament sheath has no counterpart in exoflagellated bacteria, whose filaments consist of flagellin alone [3]."

**R2.3** — *"L108-109 'one additional unidentified component, distributed with a radially asymmetric organization and a ~52 Å periodicity along the filament (Fig. 1b,d).' is it the sheath or the unidentified component that is radially asymmetric and 52 Å periodicity? these figures do not show this."*

The phrasing was indeed ambiguous. What we intended is that the sheath is organised around the filament in a radially asymmetric fashion, and that its axial repeat matches the ~52 Å helical repeat of the FlaB core (11 × 4.73 Å = 52.0 Å, corresponding to the 11 protofilaments of the canonical bacterial flagellin lattice). The text has been split and revised: the 52 Å helical repeat is now introduced in the core-architecture sentence at line 112 (revised version), and the sheath description (lines 118-121) states that the sheath tracks this repeat

rather than introducing it as a sheath-specific feature. Fig. 1 and its legend have been annotated accordingly.

**R2.4** — *"L120 what is a 'heterogeneous organization'? you have a high resolution structure, so the organization must not be heterogeneous"*

Agreed. The sheath architecture is *asymmetric and positionally deterministic*: different sheath proteins occupy position-specific protofilament sites around the filament circumference. The text has been revised to: "*The sheath proteins bind to specific core protofilaments around the filament circumference, giving each side of the assembly distinct features (Fig. 1d;…*"

**R2.5** — *"L130-131 'density for one additional outer-tier component, capping FlaA2 and FlaAP, could not be interpreted' where is this in the figures? there should be density maps... — the only density shown is of 3 amino acids in fig 1d."*

We believe the reviewer overlooked Extended Data Figure 4 (duly referenced). Several regions of *L. biflexa* and *L. interrogans* cryoEM maps were indeed shown, focusing on the novel proteins (B0STN7, B0SMK8, B0STF2 and Q72MR9). However, we thank the reviewer for her/his suggestion to also add the region of the map corresponding to this unidentified component in *L. biflexa*. Its position relative to FlaA2 and FlaAP is marked, and the main-text reference now points the reader to this panel (Extended Data Fig. 4d) in lines 134-136.

**R2.6** — *"L145-148. It is difficult to relate the text to the figure, which discusses the structure in terms of chains A,B,C. Where is the jelly roll like beta sandwich?"*

Figure 2 has been updated to label the jelly-roll β-sandwich, the concave groove and the two convex facets directly on the ribbon model, replacing the previous chain-letter labels.

**R2.7** — *"Extended Figure 6a shows the concave and convex faces, which are not visible in Figure 2."*

A concave/convex orientation indicator has been added to the main Figure 2 panel to match Ext. Data Fig. 6a, so the two are now consistent.

**R2.8** — *"Extended Figure 6b is not interpretable."*

We thank the reviewer for pointing this out, and regret the figure was not clear. Extended Data Fig. 6b has been redone and the legend rewritten.

**R2.9** — *"L163-164 'Whether FlaA1 is incorporated within native filaments remained to be determined.' this is confusing — is L. borgpetersenii a native filament?"*

The sentence as written implied a false alternative. *L. borgpetersenii* filaments are certainly native; the point we intended to make was that FlaA1 had been characterised structurally in isolation but not yet localised within a reconstructed filament assembly. The sentence has been rewritten, also considering reviewers #1's comment: "Whether FlaA1 is incorporated as a structural component within assembled filaments, possibly occupying the unfilled groove between the two FlaA2 protofilaments, remained to be determined by direct structural analysis." The wording "native" was also avoided elsewhere for consistency.

**R2.10** — *"L170-171 please clarify 'further density modification'. Extended Data Fig 5 shows a final resolution of 4.4, not 4.2 as stated in the text."*

We thank the reviewer for prompting this clarification. The two resolution values referred to different stages of the reconstruction pipeline, before and after the final solvent-flattening step. To make this transparent, we have revised all three cryo-EM workflow figures (Extended Data Figs. 2, 5 and 8) to show the solvent-flattening step explicitly and list the resolution before and after.

**R2.11** — *"Extended Data Fig 6b — the drawing appears to indicate that each subunit rotates 45 degrees, for a total change of 90 degrees, however the text indicates the total change is 45 degrees; which is correct?"*

We thank the reviewer for pointing us towards a better presentation of these subunit relationships. Our revision of Extended Data Fig. 6b (see above) has now addressed this point.

**R2.12** — *"L245-253 — is there a way to describe what 'quantitative curvature' measures that results in units of inverse microns?"*

A new Extended Data Fig. 10 has been added illustrating the meaning of κ both in a simple planar case (where κ = 1/R, the inverse of the radius of the osculating-circle, hence the units of $\mu m^{-1}$); and in the 3D operational form actually used by the Crenshaw algorithm (κ proportional to the angle φ between successive segment vectors normalised by their total length). A clarifying sentence has been added at first use of κ in the Results referencing this figure.

We also revised this paragraph and the following one (line 254 original MS / line 273 revised MS) to highlight the correlation between increased number of seams and increased curvature, which fits with existing models of flagellar polymorphism

**R2.13** — *"Table 1 - clarify 'Exclusive' and define 'FC'."*

Done. Table 1 now defines "Exclusive" (proteins detected in one condition but below the detection threshold in the other) and "FC" (fold change, wild-type over mutant) in the table footnote.

**R2.14** — *"L190 only Fig 3b (not 3c) shows 9 distinct proteins."*

Corrected.

**R2.15** — *"L245. an explanation of what a curvature of x/micron means would be helpful."*

See reply to R2.12 above (as well as to reviewer #4 below).

**R2.16** — *"L305-306. using delta k here is confusing, as it was used as an error measurement above."*

Δκ was never meant to be used as error measurement, but rather as difference or change in median curvature between strains. To improve clarity, the first time Δκ is used in Results

(previous version line 248), we make this definition explicit. The same meaning is consistently used throughout.

Quantitative estimations of error are only given in the Methods section: we used non-parametric statistics to better handle skewed distributions, hence the use of medians and interquartile ranges (IQR) to describe central tendency and dispersion. IQR was now spelled out when referred to, in the Methods section.

**R2.17** — *"L266-268 'revealing an integrated architectural system that tunes flagellar mechanical properties essential for Leptospira motility in environments of varying viscosity.' is discussion, not results."*

Agreed. The Results sentence has been trimmed to: "Together, these observations establish that core flagellin identity is directly coupled to sheath organization, seam distribution, and filament curvature." (as a conclusion from the evidence presented). The motility-viscosity interpretation remains now only in the Discussion (under the "Viscosity-dependent motility and virulence" sub-section).

**R2.18** — *"L339-340 'This suggests that additional/alternative components may be incorporated at different lifecycle stages or at precise filament locations.' This is a stretch of what one can say from the data. To validate this, one would have to have synchronous cultures and do LC-MS at specific cell-cycle timepoints."*

We thank the reviewer for this pertinent comment, and agree that our data do not directly support this as a conclusion. The text has been revised to (i) state the observation explicitly, (ii) acknowledge that the underlying mechanism (i.e. compositional plasticity) remains to be characterised, and (iii) introduce the specific scenario of differential incorporation at lifecycle stages or specific locations, as an explicitly speculative yet plausible hypothesis worth testing. The biological rationale (modulating filament architecture in response to environmental cues) has been added to clarify why we believe the scenario is biologically interesting.

**R2.19** — *"L834 — '11 protomers from different protofilaments at one axial level' this is a helical filament, so they are not at 'one axial level'."*

Agreed; the original wording incorrectly described 11 helically-related protomers as occupying "one axial level". The Extended Data Fig. 10d legend now describes the left panel as "11 protomers from successive protofilaments, all within one ~52 Å axial repeat; if the helical symmetry were perfect these would be identical, but they exhibit substantial conformational spread." The right-panel description and the surrounding mechanistic framing are unchanged.

**R2.20** — *"L552 - were the map data collection and resulting resolution differences due solely to the dataset sizes (if this is not known, it does not need to be addressed)."*

We thank the reviewer for suggesting this explanation for the resolution differences. We agree this would neatly account for the lower resolution of the *L. interrogans* maps, but (as also anticipated by the reviewer) we do not currently have any evidence to further support it.

---

## Reviewer 3

**R3.1** — *"l. 117-119 and elsewhere. It is stated that FlaB4 is the major flagellar core component in L. biflexa, and FlaB1 is the dominant FlaB paralogue in the L. interrogans serovar Manilae strain L495 examined. It is unclear how the 'dominant' core protein(s) could be determined by cryoEM alone, in that all the structures of the closely related core proteins would be averaged unless they are in a regular arrangement. The crosslinking MS studies show that FlaB4, FlaB1, and FlaB2 were crosslinked indicating that they were in close proximity, which seems contrary to the predominant presence of a single FlaB protein. Also, it seems unlikely that FlaB3 would not be incorporated into the flagella at all. The authors have ample MS data, which should be of value in determining the relative concentrations of the FlaB paralogues in the purified flagella from the wt organisms and flaB1 L. interrogans mutant. This information should be incorporated into the manuscript and the interpretation of the core composition reevaluated accordingly."*

We thank the reviewer for this important comment. The concern is legitimate and we have substantially expanded the supporting evidence for the FlaB isoform assignments. The revised manuscript now includes a new Supplementary Table (Supp Table 4), compiling FlaB isoform quantitative mass-spectrometry data across the relevant strains, and the Results text (lines 115–117; 183-185; 240-242) has been rewritten to cite this evidence explicitly. We have generated new MS data to analyze the filaments purified from the *flaB1- L. interrogans* strain. The combined argument is as follows:

*Wild-type L. biflexa*: the label-free quantitative proteomics data that we reported in our first submission (PRIDE ID PXD072058), included *L. biflexa wt* strain (mass spectrometry of purified filaments; three biological replicates; PatternLab for Proteomics analysis). Focusing on the comparative analysis of FlaB isoforms, and consistent with what we see in the cryoEM maps, FlaB4 is the most abundant isoform at 42.3 ± 2.5% of the total FlaB signal, followed by FlaB1 (28.1 ± 2.5%) and FlaB2 (26.5 ± 2.0%), with FlaB3 at substantially lower levels (3.1 ± 0.4%). The enrichment of FlaB4 over FlaB1 and FlaB2 is statistically significant by paired t-test on per-replicate percentages ($p = 0.035$ and $p = 0.018$, respectively). These data are now presented in Block A of the new Supplementary Table 4.

*Wild-type L. interrogans*: the absolute cellular abundance of the four FlaB isoforms has been determined first by Malmström et al. (Nature 460, 762–765, 2009) using heavy-labelled reference peptides, and then confirmed by Schmidt et al. (Mol Syst Biol 7, 510, 2011). These references were already cited in our initially submitted manuscript. It is clear from such studies that the most abundant isoform in *wt L. interrogans* is FlaB1 (~70% of the total FlaB signal; followed by FlaB4 at ~21%). These data are included for reference in Block B of the new Supplementary Table 4. The identity of the dominant FlaB isoform therefore differs between the two species (FlaB1 in *L. interrogans*; FlaB4 in *L. biflexa*), even though the magnitude of dominance is more modest in *L. biflexa*.

*L. interrogans flaB1⁻ mutant*: new data have been produced to address the reviewer's comment. Label-free quantitative proteomics has been performed on purified filaments from *L. interrogans flaB1⁻* (five biological replicates; MaxQuant analysis). We show that FlaB4 is indeed the most abundant isoform, confirming what we see by cryoEM. Presented in Block C of the new Supplementary Table 4, FlaB4 accounts for ~95% of the functional FlaB pool, while FlaB2 and FlaB3 drop to ~4% and ~1% respectively. The residual FlaB1 peptides map upstream of the transposon insertion site and correspond to a truncated, non-functional

product of the disrupted gene. The underlying regulatory mechanism by which FlaB2 and FlaB3 are co-depleted in this background is under investigation and will be reported elsewhere; for the present purposes, the relevant observation is that the *flaB1⁻* cryo-EM density corresponds to an essentially FlaB4-exclusive core

The reviewer also raised two specific points that we now address directly: (i) FlaB3 is indeed detected in our filament preparations, and is not absent; it is simply at low relative abundance (~3% in *L. biflexa wt*; ~1% in both *L. interrogans wt* and *flaB1⁻*). The Results text has been modified to state this explicitly. The low abundance of FlaB3 also explains why XL-MS captured few inter-protomer cross-links involving it: the low steady-state stoichiometry yields fewer productive cross-linking events, not the absence of FlaB3 from the filament. (ii) The XL-MS observation that FlaB1, FlaB2 and FlaB4 are cross-linked to each other is entirely compatible with a core in which one isoform predominates: even at 42% of the pool, a dominant FlaB4 population contacts its minor FlaB1 and FlaB2 neighbours with high probability. The cryo-EM density thus reports the majority occupancy at each lattice position.

**R3.2a** — *"Please provide a supplemental table indicating the genome sequences used and the gene designations and PDB numbers for each of the proteins described in this manuscript. In particular, the FlaB1 through FlaB4 designations are confusing and do not appear to match prior designations. Note that a newly annotated version of the L. biflexa Patoc 1 genome was released recently (NC_010602.1)."*

NC_010602.1 is the RefSeq for chromosome I within the *Leptospira biflexa* Patoc 1 whole genome sequence data in BioProject PRJNA20133 (which also includes the other chromosome II, and stable plasmid p74). We have now included these specific details in new Supplementary Table S5 (in the first submission this was Supplementary Table 4). The new version of Supp Table 5 has been extended to contain the genome locus tags (we understand this is what the reviewer refers to when mentioning gene designations) for each of the proteins. This of course includes the four isoforms of flagellin (FlaB1-4), which due to how different publications have named them in the past, is indeed an unfortunate source of confusion.

Also relevant to point out, NC_010602.1 might have been renamed recently (we are not clear about why the reviewer states that it was recently <u>released</u>), but it clearly corresponds to the same original report by Picardeau et al (2008), which we now also included within cited references (both in Supp Table 5, as well as in Methods) to avoid any ambiguity. The same was done for *L. interrogans* (for which the WGS reference used is the one reported by Nascimento et al 2004, now duly cited).

**R3.2b** — *"It should be clearly stated in the figure legends that the designations (e.g. B0SMK8) are for PDB accession numbers, if that is the case. (They do not as yet appear in the PDB.)"*

Identifiers such as B0SMK8 are UniProt accessions (assigned when no gene name has been registered for the corresponding gene), not PDB accessions. This is stated in the legend of Figure 1, where these labels first appear, and the convention applies throughout the manuscript.

**R3.3** — *"Leptospira are different from other spirochetes, in that the flagella are quite short. It would be worthwhile to include some discussion about this aspect and how the complex*

*sheath may be important in providing in stiffening the flagella and (perhaps) promoting locomotion."*

Agreed; this point has been incorporated in the Discussion, highlighting that *Leptospira* operates a single, short filament per cell pole (notably without inter-filament overlap in the centre of the cell, in contrast to other spirochetes) followed by the closing remark that the elaborate sheath we describe plausibly contributes the bending stiffness required to transmit torque productively/efficiently under these constraints.

**R3.4** — *(There is no R3.4 in the reviewer text — the reviewer's numbering skips from 3 to 5.)*

**R3.5** — *"During swimming motion, the flagella at either end are rotating in opposite directions. Are there any theories as to how the thickness of the sheath may affect rotation in opposite directions? Did classification indicate different classes consistent with alterations in the structure?"*

We agree that filament supercoil handedness must change upon motor reversal and that this raises an interesting mechanical question. A discussion paragraph has now been added, proposing that the millisecond timescale of reversal precludes sheath compositional rearrangement, but is plausibly accommodated by seam transitions in the core, facilitated near the FlaA1 dimer pair where the absence of direct core-sheath contacts (and the apparent plasticity of the FlaA1–FlaA1 interfaces) would relieve the mechanical constraints that elsewhere lock the sheath onto the core.

On the specific question of whether classification revealed structural alterations: 3D classification during cryo-EM processing definitely identified numerous structural sub-populations, some of which are visible in the illustrated 2D and 3D classification steps of our cryo-EM workflow Extended Data Figures. However, these sub-populations seemed to represent various stages of sheath dis/assembly, and classifications were evidently not clean enough to do in-depth structural analysis. Within the most completely assembled sheath populations, corresponding to the structures reported in this manuscript, we did not resolve discrete conformational sub-populations. Given that substantial sub-populations of different FlaB isoforms were present in our wild-type samples, we suspect that structural sub-populations were indeed present, but not detectable due to limitations in data collection and methods. This could be a fruitful topic for future investigation.

**R3.6** — *"Fig. 1 and associated text. The absence of FlaA1 from the L. biflexa filament structure may simply reflect that this association is less stable than in L. interrogans, resulting in its loss during purification."*

We agree. FlaA1 is detected by mass spectrometry in *L. biflexa* filament preparations, including clear FlaA2 crosslinks (Fig. 1c; Table 1), so it is present after purification; the most likely interpretation is that the FlaA1-filament association is weaker in *L. biflexa* than in *L. interrogans*, and dissociates progressively during sample manipulation. The Results text has been amended accordingly.

**R3.7** — *"l. 58 (minor). estimated to cause?"*

Corrected.

## Reviewer 4

**R4.1** — *"Given the importance of 'curvature' in this paper, it was difficult as a non-specialist to determine the exact physical meaning of curvature as they use it. The axis of the relevant plots is labeled in units of $\mu m^{-1}$, so I assume it is something per micron. Is it superhelical waves per micron? A cartoon would help. The movies are not quite clear enough to see the superhelical corkscrew shape."*

This concern is shared with Reviewer 2 (R2.12, R2.15) and has been addressed with a new cartoon and an explanatory sentence at first use.

A new Extended Data Fig. 10 has been added illustrating the meaning of κ both in a simple planar case (where $\kappa = 1/R$, the inverse of the radius of the osculating-circle, hence the units of $\mu m^{-1}$); and in the 3D operational form actually used by the Crenshaw algorithm (κ proportional to the angle φ between successive segment vectors normalised by their total length). A clarifying sentence has been added at first use of κ in the Results referencing this figure.

## Miscellaneous corrections

(line numberings of the revised manuscript are provided)

Line 911 (Extended Data Figure 8) and 918-920 (legend): Fourier Shell Correlation graphs of the three 3D maps have been added as a new panel in Extended Data Fig. 8. A corresponding text reference (line 640) was also added in Methods.

Line 671: Incorrect pixel size values were fixed: '(3.39 Å for ….for L. interrogans flaB1-)' -> '(0.534 Å for all three data sets)'

Line 985 (within Supplementary Table 1): 'Pixel size (Å)' -> 'Physical pixel size (Å)'
